# Supplementary material for: MLG: multilayer graph clustering for multi-condition scRNA-seq data
Source: Nucleic Acids Res. 2021 Sep 28;49(22):e127. doi: 10.1093/nar/gkab823 (PMC8682753; doi:10.1093/nar/gkab823)
Supplement: gkab823_Supplemental_File [file gkab823_supplemental_file.pdf]

Supplementary materials for "MLG: Multilayer graph clustering for multi-condition scRNA-seq data"

Shan Lu[1], Daniel J. Conn[2], Shuyang Chen[1], Kirby D. Johnson[3],
Emery H. Bresnick[3], and Sündüz Keleş[1,2]

[1] Department of Statistics, University of Wisconsin, Madison, WI, USA.

[2] Department of Biostatistics and Medical Informatics, University of Wisconsin School of Medicine and Public Health, Madison, WI, USA.

[3] Wisconsin Blood Cancer Research Institute, Department of Cell and Regenerative Biology, University of Wisconsin School of Medicine and Public Health, Madison, WI, USA.

1 Supplementary materials

1.1 Aggregation of multiple SNNs boost signal-to-noise ratio

To investigate the impact of aggregating SNNs from multiple low-dimensional embeddings, we first define "signal-to-noise ratio" of a graph. We base our analysis on stochastic block models (SBMs) [1] which are generative random graph models that serve as canonical models for investigating graph clustering and community detection. In the view of SBMs, cells are vertices of a graph and clusters depicting cell types represent distinct communities. We denote the total number of cells (vertices) by n, the number of communities (clusters) by K. Let σ be a cluster assignment function where $\sigma(i)$ corresponds to the cluster label for cell i, and $\theta_{\sigma(i)\sigma(j)}$ denote the connectivity probability (i.e., edge probability) between cells i and j. Furthermore, let θ_{in} and θ_{out} denote the minimum in-cluster and maximum out-of-cluster connectivity probability. We denote the overall

parameter space as

$$\begin{aligned}\Theta(n, K, \theta_{in}, \theta_{out}, \beta) =\Big\{ & \big(\sigma, \{\theta_{k\ell}\}_{k,\ell=1,\dots,K}\big) : \sigma \in [K]^n, \\ & n_k \in \Big[\frac{n}{\beta K}, \frac{\beta n}{K}\Big], \forall k \in [K], \\ & \{\theta_{kl}\} \in [0,1]^{K\times K}, \text{ and} \\ & \theta_{kk} \geq \theta_{in}, \\ & \theta_{k\ell} \leq \theta_{out} \text{ if } k \neq \ell \Big\},\end{aligned} \tag{1}$$

where n_k denotes the size of cluster k, $k = 1, \cdots, K$ and $\beta \geq 1$ is a constant setting a lower bound for minimum cluster size.

For a cluster assignment $\hat{\sigma}$, the mismatch rate, which quantifies the discrepancy between the true cluster labels and the assignments from $\hat{\sigma}$, is defined in a way that accounts for equivalent partitions

$$r(\sigma, \hat{\sigma}) := 1 - \max_{\pi} \frac{1}{n} \sum_{i=1}^{n} \mathbf{1}\{\sigma(i) = \pi(\hat{\sigma})(i)\}, \tag{2}$$

where $\mathbf{1}$ is an indicator function, π is a permutation of cluster labels of n vertices, and the function $\mathbf{1}\{\sigma(i) = \pi(\hat{\sigma})(i)\}$ returns 1 if the cluster assignment for cell i is correct under a clustering (i.e., partition) $\pi(\hat{\sigma})$ which is equivalent to $\hat{\sigma}$. Zhang et al. [2] showed that the minimax convergence of mismatch rate as $\frac{nI}{K \log K} \to \infty$ is

$$\inf_{\hat{\sigma}} \sup_{\Theta(n,K,\theta_{in},\theta_{out},\beta)} \mathbb{E}r(\sigma, \hat{\sigma}) = \begin{cases} \exp\left\{-(1+o(1))\frac{nI}{2}\right\}, & K = 2, \\ \exp\left\{-(1+o(1))\frac{nI}{\beta K}\right\}, & K \geq 3, \end{cases} \tag{3}$$

where $1 + \epsilon_n \leq \beta < \sqrt{5/3}$ for some $\epsilon_n = CK/n$ with a large enough constant C. Here, I is a constant related to minimum in-cluster and maximum out-of-cluster connectivity probabilities θ_{in} and θ_{out} and is defined as

$$I := -2 \log\left(\sqrt{\theta_{in}\theta_{out}} + \sqrt{(1-\theta_{in})(1-\theta_{out})}\right). \tag{4}$$

Furthermore, Zhang et al. [2] showed that I is asymptotically equivalent to

$$\tilde{I} := \frac{(\theta_{in} - \theta_{out})^2}{\theta_{in}}. \tag{5}$$

$\tilde{I}$ can be interpreted as the "signal-to-noise ratio" of the graph. A larger $\tilde{I}$, hence I, leads to a faster convergence of mismatch rate to 0. We utilize this notion of "signal-to-noise ratio" to investigate the impact of aggregation. Specifically, given adjacency matrices A_1 and A_2 of two independent SBM graphs on the same set of cells, we use superscript to indicate their respective parameters, e.g., $\theta_{k\ell}^{A_1}$ represents the connectivity probability of cells in cluster k and cells in cluster ℓ in graph A_1. Next, we define the adjacency matrix of "union graph" B as

$$B_{i,j} := \begin{cases} 1, & \text{if } (A_1)_{ij} = 1 \text{ or } (A_2)_{ij} = 1, \\ 0, & \text{o.w.}, \end{cases} \tag{6}$$

where $(A_1)_{ij}$ denotes the entry at the i-th row and j-th column of the adjacency matrix A_1. Then, $\theta_{k\ell}^B$, the connectivity probability of cells in cluster k and cells in cluster ℓ in the union graph is given by

$$\begin{aligned} \theta_{k\ell}^B :=& \mathbb{P}\Big(B_{i,j} = 1 \Big| \sigma(i) = k, \sigma(j) = \ell\Big) \\ =& 1 - \mathbb{P}\Big((A_1)_{i,j} = 0 \text{ and } (A_2)_{i,j} = 0 \Big| \sigma(i) = k, \sigma(j) = \ell\Big) \\ =& 1 - \mathbb{P}\Big((A_1)_{i,j} = 0 \Big| \sigma(i) = k, \sigma(j) = \ell\Big) \times \\ & \mathbb{P}\Big((A_2)_{i,j} = 0 \Big| \sigma(i) = k, \sigma(j) = \ell\Big) \\ =& 1 - (1 - \theta_{k\ell}^{A_1})(1 - \theta_{k\ell}^{A_2}) \\ =& \theta_{k\ell}^{A_1} + \theta_{k\ell}^{A_2} - \theta_{k\ell}^{A_1}\theta_{k\ell}^{A_2}. \end{aligned} \tag{7}$$

The resulting union graph has the stochastic block structure with minimum in-cluster connectivity $\theta_{in}^B \geq \theta_{in}^{A_1} + \theta_{in}^{A_2} - \theta_{in}^{A_1}\theta_{in}^{A_2}$, and maximum out-of-cluster connectivity $\theta_{out}^B \leq \theta_{out}^{A_1} + \theta_{out}^{A_2} - \theta_{out}^{A_1}\theta_{out}^{A_2}$.

Let $\tilde{I}^{A_1}$, $\tilde{I}^{A_2}$ and $\tilde{I}^B$ denote the signal-to-noise ratios of graphs A_1, A_2, and B, respectively.

Then, we have

$$
\begin{aligned}
\tilde{I}^B =& \frac{(\theta_{in}^B - \theta_{out}^B)^2}{\theta_{in}^B} \\
\geq & \frac{(\theta_{in}^{A_1} + \theta_{in}^{A_2} - \theta_{in}^{A_1}\theta_{in}^{A_2} - \theta_{out}^{A_1} - \theta_{out}^{A_2} + \theta_{out}^{A_1}\theta_{out}^{A_2})^2}{\theta_{in}^{A_1} + \theta_{in}^{A_2} - \theta_{in}^{A_1}\theta_{in}^{A_2}}.
\end{aligned}
\tag{8}
$$

Under $\theta_{in}^{A_1} = \theta_{in}^{A_2}$ and $\theta_{out}^{A_1} = \theta_{out}^{A_2}$, we have

$$
\begin{aligned}
\tilde{I}^B \geq & \frac{(2 - \theta_{in}^{A_1} - \theta_{out}^{A_1})^2}{2 - \theta_{in}^{A_1}} \times \frac{(\theta_{in}^{A_1} - \theta_{out}^{A_1})^2}{\theta_{in}^{A_1}} \\
=& \tilde{I}_{A_1} \times \frac{(2 - \theta_{in}^{A_1} - \theta_{out}^{A_1})^2}{2 - \theta_{in}^{A_1}}.
\end{aligned}
\tag{9}
$$

This relation implies that when A_1, A_2 are very sparse, i.e., with small $\theta_{in}^{A_1}$ and $\theta_{out}^{A_1}$, the signal-to-noise ratio $\tilde{I}_B$ of the union graph is almost twice as large as the individual graphs $\tilde{I}_{A_1}$ and $\tilde{I}_{A_2}$.

1.2 Dimension reduction perturbs graph neighbors

We analyzed the general impact of dimension reduction on the local neighborhoods of the cells under a simple model where data for each cell is from a mixture of Gaussian distributions with $K+1$ components. Specifically, let y_0, y_i, and y_j denote the expression vectors of three cells drawn from the Gaussian mixture $\sum_{k=0}^{K} p_k \mathcal{N}(\mu_k, \sigma^2 I_d)$, where $\mathcal{N}(.,.)$ denotes the Gaussian probability density function. Here, $K+1$ is the number of clusters, i.e., cell types, d is the dimension of the data from the Gaussian mixture, i.e., numbers of genes, p_k's with $\sum_{k=0}^{K} p_k = 1$ are the mixing probabilities of the mixture distribution and represent the population proportion of each cell type. Without loss of generality, suppose a cell indexed by 0 and with expression profile y_0 is from cluster 0, and cells i and j with expression profiles y_i, y_j are from cluster 1. Next, we consider projecting the d-dimensional (d very large) y_0, y_i, and y_j onto a d_0-dimensional space (d_0 very small) and investigate the relative ordering of the distances between cells i, j to cell 0 in the d_0-dimensional space to their orderings in the d-dimensional space.

The following lemma shows diminishing dependence between these two sets of orderings which

implies low levels of edge overlap between the shared nearest neighbor graph in the low-dimensional space and the shared nearest neighbor graph constructed from the original high dimensional data.

Lemma 1.1. *Suppose* $y_i, y_j \in \mathcal{N}(\mu_1, \sigma^2 I_d)$, $y_0 \in \mathcal{N}(\mu_0, \sigma^2 I_d)$, $Z = [z_1, \ldots, z_{d_0}] \in \mathbb{R}^{d \times d_0}$, *where* $\{z_t\}_{t=1,\ldots,d_0}$ *are linearly independent unit vectors. Define* $u_t = (Z^T Z)^{-1} Z^T y_t$ *for* $t = 0, i, j$. *If* d_0 *is fixed,* $\|\mu_1 - \mu_0\|_4$ *is bounded as* $d \to \infty$, *then we have*

$$\Big| \mathbb{P}\left[\|y_i - y_0\|^2 > \|y_j - y_0\|^2, \|u_i - u_0\|^2 > \|u_j - u_0\|^2\right] - \mathbb{P}\left[\|y_i - y_0\|^2 > \|y_j - y_0\|^2\right] \mathbb{P}\left[\|u_i - u_0\|^2 > \|u_j - u_0\|^2\right] \Big| \to 0$$

Proof. Define $H = Z(Z^T Z)^{-1} Z^T$ as the projection matrix onto d_0 dimensional space, and the orthogonal complement of H as $H^\perp = I - H$.

$$\begin{aligned} \mathbb{E}\Big| \|H(y_i - y_0)\|^2 - \|H(y_j - y_0)\|^2 \Big| &\leq 2\mathbb{E}\|H(y_i - y_0)\|^2 && (10) \\ &= 4d_0\sigma^2 + 2(\mu_1 - \mu_0)^T H(\mu_1 - \mu_0) && (11) \\ &\leq 4d_0\sigma^2 + 2\|\mu_1 - \mu_0\|^2. && (12) \end{aligned}$$

By Markov's inequality, for any $t > 0$,

$$\mathbb{P}\Big\{ \Big| \|H(y_i - y_0)\|^2 - \|H(y_j - y_0)\|^2 \Big| \leq t \Big\} \geq 1 - \frac{4d_0\sigma^2 + 2\|\mu_1 - \mu_0\|^2}{t}. \tag{13}$$

Suppose the eigenvalue decomposition of projection matrix $H^\perp$ has the form $H^\perp = U I_{d-d_0} U^T$. Define $v_t = U^T(y_t - y_0)$, for $t = i, j$, and $\tilde{\mu} = U^T(\mu_1 - \mu_0)$. Then $v_{tk} \sim \mathcal{N}(\tilde{\mu}_k, 2\sigma^2)$ and v_{tk} are mutually independent for $t = i, j$ and $k = 1, \ldots, d - d_0$, and

$$\|H^\perp(y_i - y_0)\|^2 - \|H^\perp(y_j - y_0)\|^2 = \sum_{k=0}^{d-d_0} (v_{i,k}^2 - v_{jk}^2), \tag{14}$$

$$\mathbb{E}\left[\frac{v_{ik}^2 - v_{jk}^2}{\sigma^2}\right] = 0, \ \mathrm{Var}\left(\frac{v_{ik}^2 - v_{jk}^2}{\sigma^2}\right) = 64 + 32\Big(\frac{\tilde{\mu}_k}{\sigma}\Big)^2. \tag{15}$$

Taking $s_d^2 = 32\sum_{k=1}^{d-d_0}[2 + \left(\frac{\tilde{\mu}_k}{\sigma}\right)^2] = 64(d - d_0) + 32\frac{(\mu_1-\mu_0)^T H^\perp(\mu_1-\mu_0)}{\sigma^2}$, we can verify the Feller's

condition [3],

$$\lim_{d\to\infty}\sum_{k=1}^{d-d_0}\frac{\mathbb{E}[\frac{v_{ik}^2-v_{jk}^2}{\sigma^2}]^4}{\epsilon^2 s_d^4}=\frac{\sum_k 3072\big[(\frac{\tilde{\mu}_k}{\sigma})^4+12(\frac{\tilde{\mu}_k}{\sigma})^2+12\big]}{\epsilon^2\big[64(d-d_0)+32\frac{(\mu_1-y_0)^T H^\perp(\mu_1-y_0)}{\sigma^2}\big]^2}\to 0, \tag{16}$$

for any $\epsilon > 0$ and $d\to\infty$, and apply the Lindeberg central limit theorem [3] to get

$$\sum_k \frac{v_{ik}^2-v_{jk}^2}{\sigma^2 s_d}\to^d \mathcal{N}(0,1). \tag{17}$$

This asymptotic result leads to the following probability bound

$$\mathbb{P}\Big(\Big|\|H^\perp(y_i-y_0)\|^2-\|H^\perp(y_j-y_0)\|^2\Big|>t\Big) \tag{18}$$

$$=\mathbb{P}\Big(\frac{|\sum_{k=1}^{d-d_0}(v_{ik}^2-v_{jk}^2)|}{\sigma^2 s_d}>\frac{t}{\sigma^2 s_d}\Big) \tag{19}$$

$$=2\left(1-\Phi\left(\frac{t}{\sigma^2 s_d}\right)\right)-o(1) \tag{20}$$

$$=1-o(1), \tag{21}$$

as $d\to\infty$. Finally, we have

$$\frac{1}{4}\quad \geq \mathbb{P}\Big[\|y_i-y_0\|^2>\|y_j-y_0\|^2, \|u_i-u_0\|^2<\|u_j-u_0\|^2\Big] \tag{22}$$

$$\geq \mathbb{P}\Big[\|H^\perp(y_i-y_0)\|^2-\|H^\perp(y_j-y_0)\|^2\geq t, \tag{23}$$

$$\Big|\|H(y_i-y_0)\|^2-\|H(y_j-y_0)\|^2\Big|<t, \|u_i-u_0\|^2<\|u_j-u_0\|^2\Big]$$

$$=\mathbb{P}\Big[\|H^\perp(y_i-y_0)\|^2-\|H^\perp(y_j-y_0)\|^2\geq t\Big] \tag{24}$$

$$\times\mathbb{P}\Big[\Big|\|H(y_i-y_0)\|^2-\|H(y_j-y_0)\|^2\Big|<t, \|u_i-u_0\|^2<\|u_j-u_0\|^2\Big]$$

$$\geq\left[\frac{1}{2}-o(1)\right]\times\left[\frac{1}{2}-o(1)\right] \tag{25}$$

$$=\frac{1}{4}-o(1) \tag{26}$$

The last inequality holds with an appropriate choice of t. □

1.3 Weighted MLG

In this section, we describe a strategy of evaluating and selecting low-dimensional embedding methods for the construction of MLG. The practical implication of this is that as new low-dimensional embedding approaches emerge, users will be able to adaptively select the layers to utilize in MLG.

Denote the normalized expression matrix as $\boldsymbol{E} \in \mathbb{R}^{G \times N}$, where N is the number of cells and G is the number of genes. Suppose we have knn graphs constructed from S low-dimensional embeddings $L_1, L_2, \ldots, L_S$. Denote the set of neighbors of cell i, $i \in \{1, 2, \ldots, N\}$, in knn($L_s$) as $\mathcal{A}_{i,L_s}$. We develop two metrics as (i) M_{L_s} to measure how well the low-dimensional embedding L_s approximates the original gene expression, and (ii) B_{L_s} to measure how well L_s removes condition effects. Specifically, M_{L_s} for low-dimensional embedding s is defined based on the squared error loss as:

$$M_{L_s} = \frac{1}{N} \sum_{i=1}^{N} \min_{j \in \mathcal{A}_{i,L_s}} \|\boldsymbol{e}_i - \boldsymbol{e}_j\|^2, \tag{27}$$

where $\boldsymbol{e}_i$ is the ith column of matrix $\boldsymbol{E}$, i.e. the vector of gene expression of cell i. For each cell i, we use the neighbor that provides the minimum squared error, because the cells in set $\mathcal{A}_{i,L_s}$ can be in different conditions from cell i, hence having large gene expression difference due to condition effects.

$$B_{L_s} = \frac{1}{K} \sum_{j \in \mathcal{A}_{i,L_s}} \mathbf{1}_{\{d_i \neq d_j\}}, \tag{28}$$

where d_i is the condition label of cell i, K is the number of neighbors in the knn graph. We use these two metrics to construct weights for each low-dimensional embedding. First, we filter out embeddings that do not perform better than the background. Specifically, we generate a background value for M_{L_s}, denoted as M^{bg}, as:

$$M^{bg} = \frac{1}{N} \sum_{i=1}^{N} \min_{j \in \mathcal{A}_i^{bg}} \|\boldsymbol{e}_i - \boldsymbol{e}_j\|^2, \tag{29}$$

where $\mathcal{A}_i^{bg}$ is randomly sampled. M^{bg} quantifies the squared error loss when the knn graph is

random. Then, we conduct a t-test with the following null hypothesis:

$$M_{L_s} \geq M^{bg}.$$

If the resulting p-value $p_{L_s} > \delta_0$, we exclude L_s in the construction of weighted MLG. Next, we define the weights as follows. Let $x_{L_s} = 1 - \frac{M_{L_s}}{M^{bg}}$ and define $u_{L_s} = x_{L_s} \times \mathbf{1}_{\{\max_s x_{L_s} > \delta_1\}} + \mathbf{1}_{\{\max_s x_{L_s} \leq \delta_1\}}$. This definition assigns equal contribution to each candidate embedding if none of the knns provides a large improvement over a random graph in terms of squared error loss. Similarly, let $v_{L_s} = B_{L_s} \times \mathbf{1}_{\{\max_s B_{L_s} > \delta_2\}} + \mathbf{1}_{\{\max_s B_{L_s} \leq \delta_2\}}$. Finally, the weight for low-dimensional embedding L_s is defined as

$$w_{L_s} = u_{L_s} \times v_{L_s} \times \mathbf{1}_{\{p_{L_s} < \delta_0\}}. \tag{30}$$

Consequently, the adjacency matrix for weighted MLG is defined as

$$A_{MLG} = \sum_{s=1}^{S} w_{L_s} A_{L_s}, \tag{31}$$

where A_{L_s} denote the adjacency matrix of the SNN graph constructed with L_s. Across the wide variety of simulation settings we have considered, we set $\delta_0 = 1e-5, \delta_1 = 0.01, \delta_2 = 0.05$.

1.4 Application of MLG to SNARE-seq data

SNARE-seq generates joint profiles of accessible chromatin (snATAC-seq) and RNA (snRNA-seq). We used the peak matrices and expression matrices for the first two replicates of neonatal and adult mouse brain cortex provided along with the raw data. Fragment files for integration were extracted from the raw snATAC-seq data using the python package `Sinto`. Following the Seurat tutorial, functions `FindIntegrationAnchors` and `IntegrateData` were applied to peak matrices with fragment files to integrate chromatin accessibility data across conditions.

We used Latent Semantic Indexing [4] (LSI) as the dimension reduction method for snATAC-seq data and removed the first LSI component from downstream analysis since it often captures sequencing depth rather than biological variation. SnRNA-seq data was processed with Seurat and

Liger for integration and further dimension reduction. Each pair of low-dimensional embeddings of snATAC-seq and snRNA-seq (LSI-Seurat, LSI-Liger) were combined to generate a weighted nearest neighbor (WNN) graph [5]. These resulting graphs were then inputted to MLG (Supplementary Figure S24A).

We compared the MLG clustering results with those of individual WNN graph clustering in terms of separability, as measured by the average silhouette score, and algorithmic stability, as measured by the variation of silhouette score with different starting values in the Louvain algorithm. Taking into account the major cell types, i.e., neurons, endothelial cells, ependymal cells, oligodendrocytes, microglia and astrocytes, in the mouse brain, we expected to detect at least six clusters when maximizing the average silhouette score. While the separability of WNN(Liger, LSI) is slightly higher than those of WNN(Seurat, Liger) and MLG, MLG yields more stable clusters, with a silhouette score invariable to the varying numbers of starting values (Supplementary Figures S24, S25 C).

Next, we sought to align MLG clusters to known cell types by leveraging the expression of cell type marker genes. Cells in cluster 1, have high expression in *Meg3* [6], *Snhg11* [7] and *Syt1* [8], which are marker genes for neurons. Cluster 2 is identified to be neuroblast by the expression of *Igfbpl1* [9]. Cells in cluster 5 show an expression pattern consistent with astrocytes based on marker genes *Aldoc* and *Slc1a3* [8]. The other three clusters are formed by subtypes of neurons: cluster 3 and 4 are enriched for *Tle4* and *Crmp1* expression, respectively, suggesting different kinds of excitatory neurons [6]; cluster 6 expresses *Nxph1*, representing inhibitory neurons [10]. The results for cluster specific gene expression is mixed in replicates 1 and 2. For replicate 1, marker gene heatmap (Supplementary Figure S24D), MLG clusters exhibit more distinguished cluster specific gene expression (Supplementary Table S5), while for replicate 2, marker gene heatmap (Supplementary Figure S25D), WNN(Liger, LSI) clusters exhibit more distinguished cluster specific gene expression (Supplementary Table S6). Collectively, this analysis supports that while MLG does not result in immediate gains to the analysis of this multi-modal data, it is readily applicable with a comparable performance to methods specific for this data type.

1.5 Computational resources required by MLG

The MLG workflow contains two steps: (1) construction of multiple low-dimensional embeddings, (2) construction of the MLG graph followed by Louvain clustering. We evaluated the computational efficiency on 4 datasets of sizes 1e3, 1e4, 5e4, and 1e5 cells that are subsampled from *Kowalczyk_1*, *Johnson_20*, and *Census_of_Immune_cells*. We provide CPU time, elapsed time, and memory usage of dimension reduction methods (PCA, cNMF, Seurat, Liger, Harmony, scVI, and scAlign) that might be utilized in step (1) and step (2) of MLG (graph construction and Louvain clustering) in Figure S26. Among the dimension reduction methods cNMF and scVI are the most time consuming in terms of CPU time. Since some of the programs are implemented in a parallel manner, we also report their elapsed times. Seurat, scVI, and cNMF take more than an hour to complete the computation and require more than 35 GB of memory for the large dataset with 100,000 cells. Step (2) "construction of the MLG graph followed by Louvain clustering" of MLG takes only 11 seconds and 0.57 GB for the dataset with 100,000 cells. The computational resources required for the MLG workflow highly depends on the choices of dimension reduction/integration methods. The computation time of MLG is approximately equal to the most time consuming dimension reduction/integration method when step (1) is implemented in parallel. The run time and memory usage of each method are measured using the "time(1)" function available in Linux systems. All jobs were run on a Linux server with 2 Intel CM8066002031501 Xeon E5-2680V4 2.40GHz 14-core Processor, 252 GB of RAM, and 8 GB of swap memory.

1.6 Supplementary figures

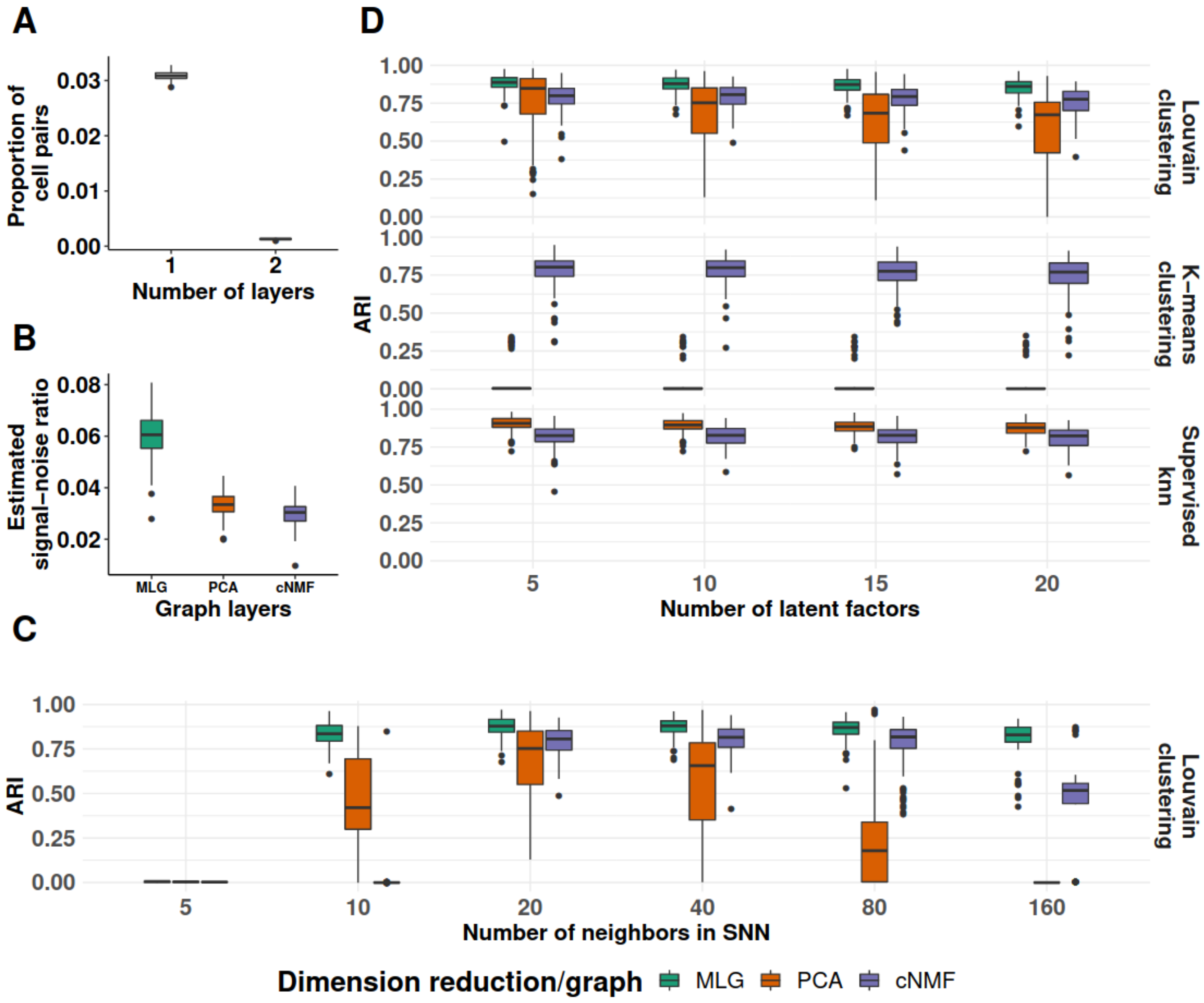

Figure S1: **Simulation results for simulation setting 2 "balanced cell type and small-condition-effect".** (A) Proportions of cell pairs with edges across different numbers of layers of MLG constructed from SNN graphs of PCA and cNMF, Seurat, and Liger (with 20 neighbors in SNN graphs and 10 latent factors in low-dimensional embeddings). The boxplots depict the proportions across all the simulation replicates. (B) Estimated signal-to-noise ratios of SNN graphs constructed from different low-dimensional embeddings and their multilayer graph across all the simulation replicates (with 20 neighbors in SNNs and 10 latent factors in low-dimensional embeddings). (C) Louvain clustering accuracy of SNN graphs as a function of numbers of neighbors in SNN graph construction (with 10 latent factors in low-dimensional embeddings). (D) Adjusted Rand index comparison of Louvain and k-means clustering of SNN graphs from different low-dimensional embeddings and their MLG as a function of number of latent factors in the low-dimensional projections (with 20 neighbors in SNN graphs). ARI values of supervised knn classifiers for individual SNN graphs are provided as reference.

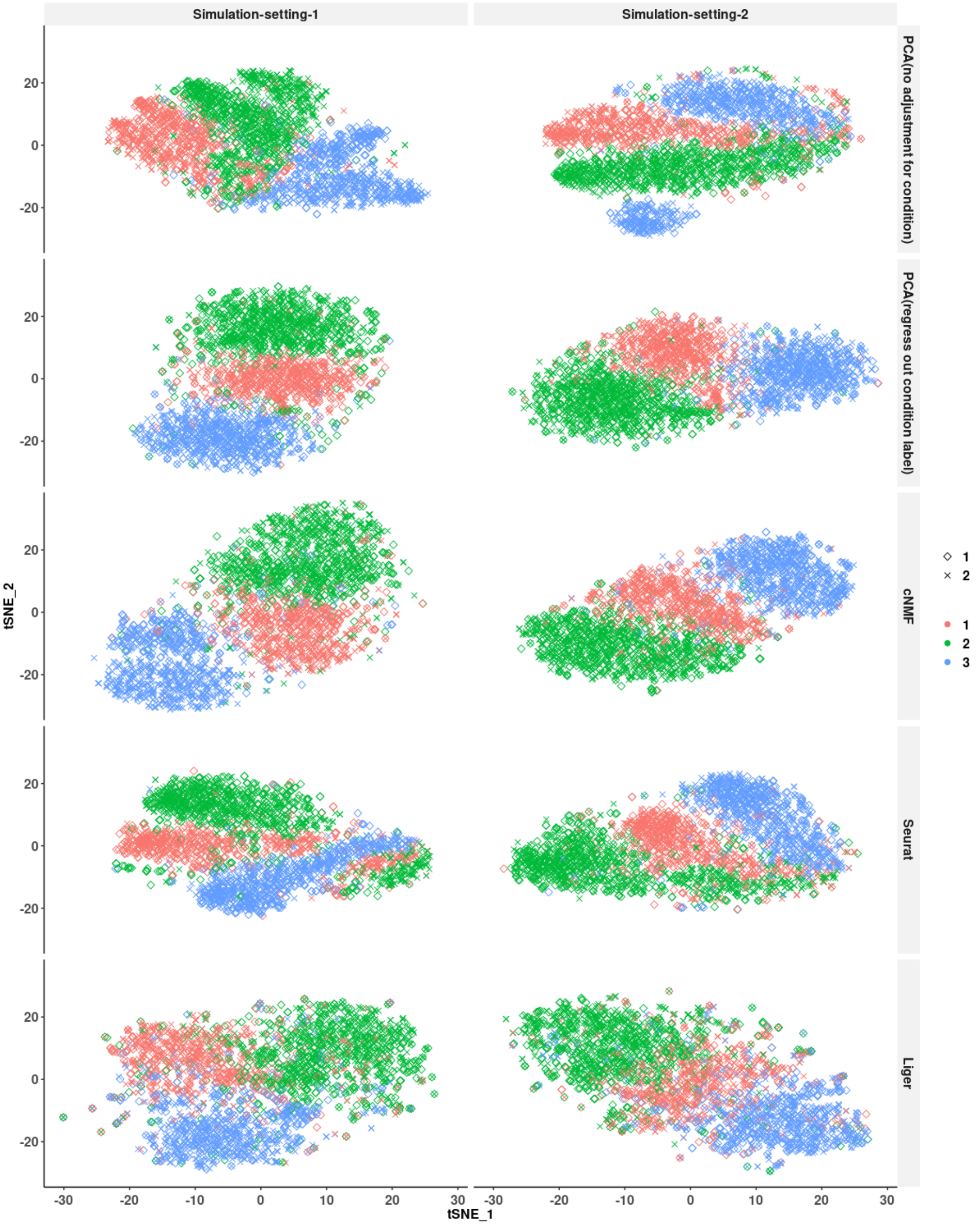

Figure S2: Balanced cell type simulation with the simulation procedure in the "Simulation" section (settings 1 and 2). Visualization of a sample simulated dataset under the tSNE coordinates computed using multiple dimension reduction results (PCA, cNMF, Seurat, Liger). Simulation-setting-1 on the left column represents "balanced cell type and large condition effects". Simulation-setting-2 on the right column represents "balanced cell type and small condition effects". Compared to the first row, the second row had an extra step in the preprocessing stage, which is to regress out unwanted variation, e.g. the condition label.

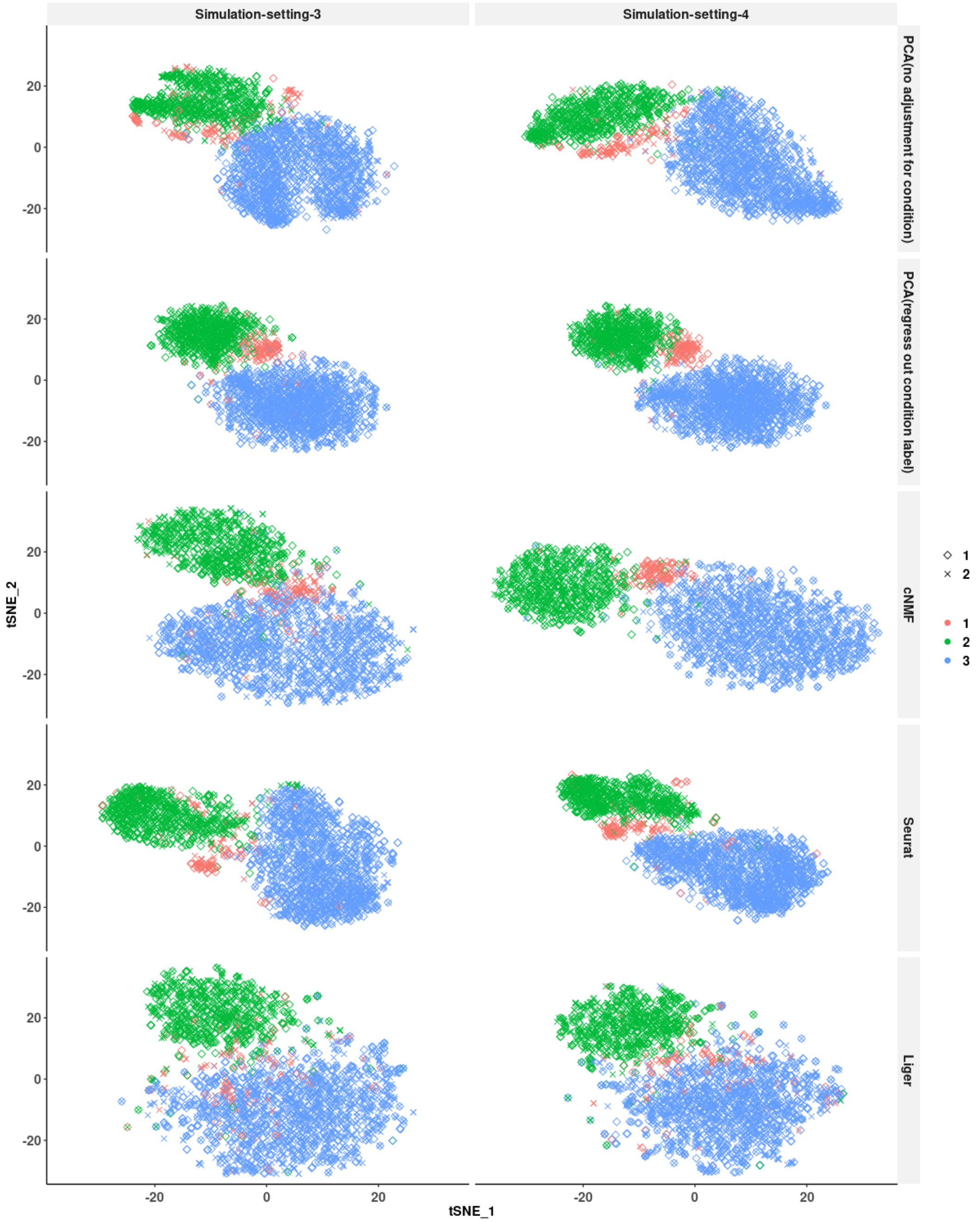

Figure S3: Rare cell type simulation with the simulation procedure described in the “Simulation” section (settings 3 and 4). Visualization of a sample simulated dataset under the tSNE coordinates computed using multiple dimension reduction results (PCA, cNMF, Seurat, and Liger). Simulation-setting-3 on the left column represents “rare cell type and large condition effects”. Simulation-setting-4 on the right column represents “rare cell type and small condition effects”. Compared to the first row, the second row had an extra step in the preprocessing stage, which is to regress out unwanted variation, e.g. the condition label.

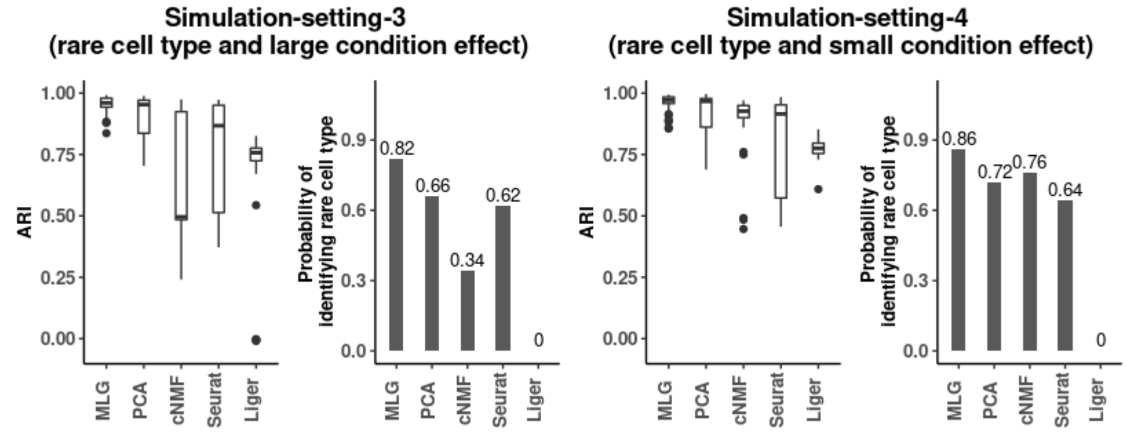

Figure S4: Clustering performance in simulation settings 3 and 4. Adjusted rand index (ARI) and rare cell type identification performances across 50 simulation replicates in simulation settings 3 and 4. PCA, cNMF, Seurat, and Liger represent Louvain clustering with the low-dimensional embeddings derived from each method. A 4-layer MLG (PCA, cNMF, Seurat, Liger) is implemented in the large condition effect setting, a 2-layer MLG (PCA, cNMF) is implemented in the small condition effect setting.

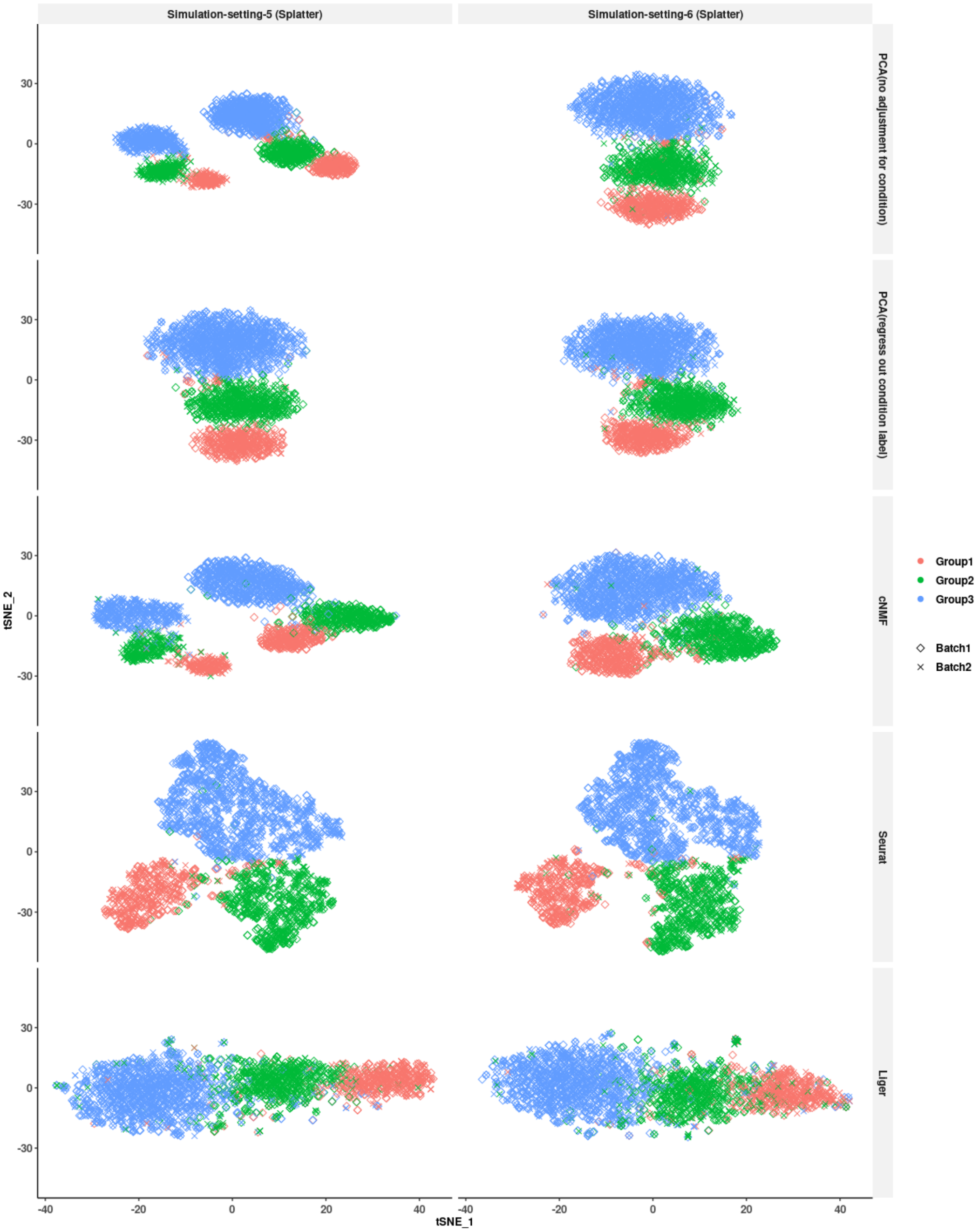

Figure S5: Visualization of a sample Splatter simulated dataset under the tSNE coordinates computed using multiple dimension reduction results (PCA, cNMF, Seurat, and Liger). "Simulation-setting-5" on the left column represents "large condition effects". "Simulation-setting-6" on the right column represents setting "small condition effects". Compared to the first row, the second row had an extra step in the preprocessing stage, which is to regress out unwanted variation, e.g. the condition label.

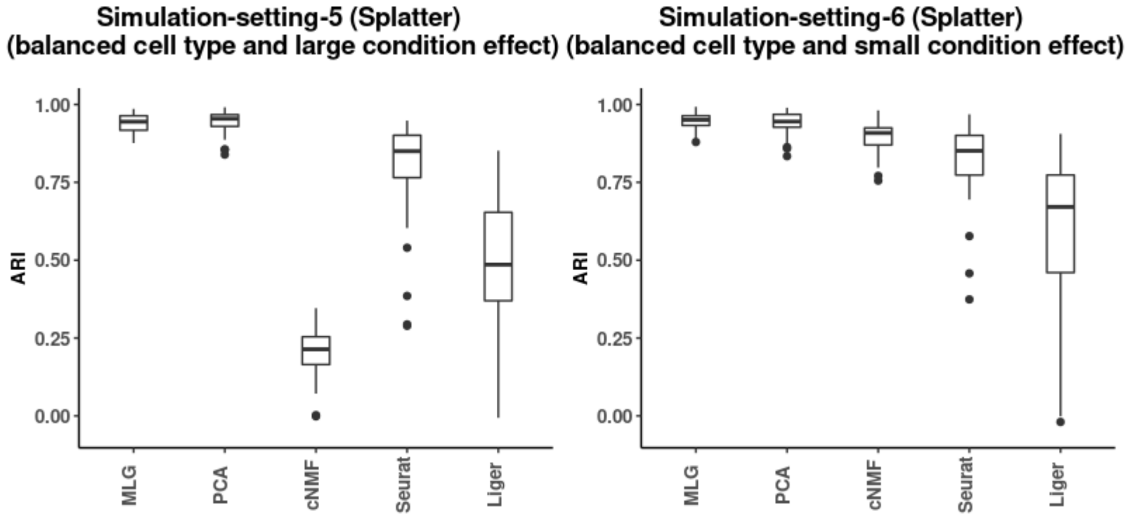

Figure S6: ARIs of clustering results across 50 Splatter simulated datasets in settings 5 and 6. PCA, cNMF, Seurat, and Liger represent Louvain clustering with the low-dimensional embeddings derived from each method. A 4-layer MLG (PCA, cNMF, Seurat, Liger) is implemented in the large condition effect setting, a 2-layer MLG (PCA, cNMF) is implemented in the small condition effect setting.

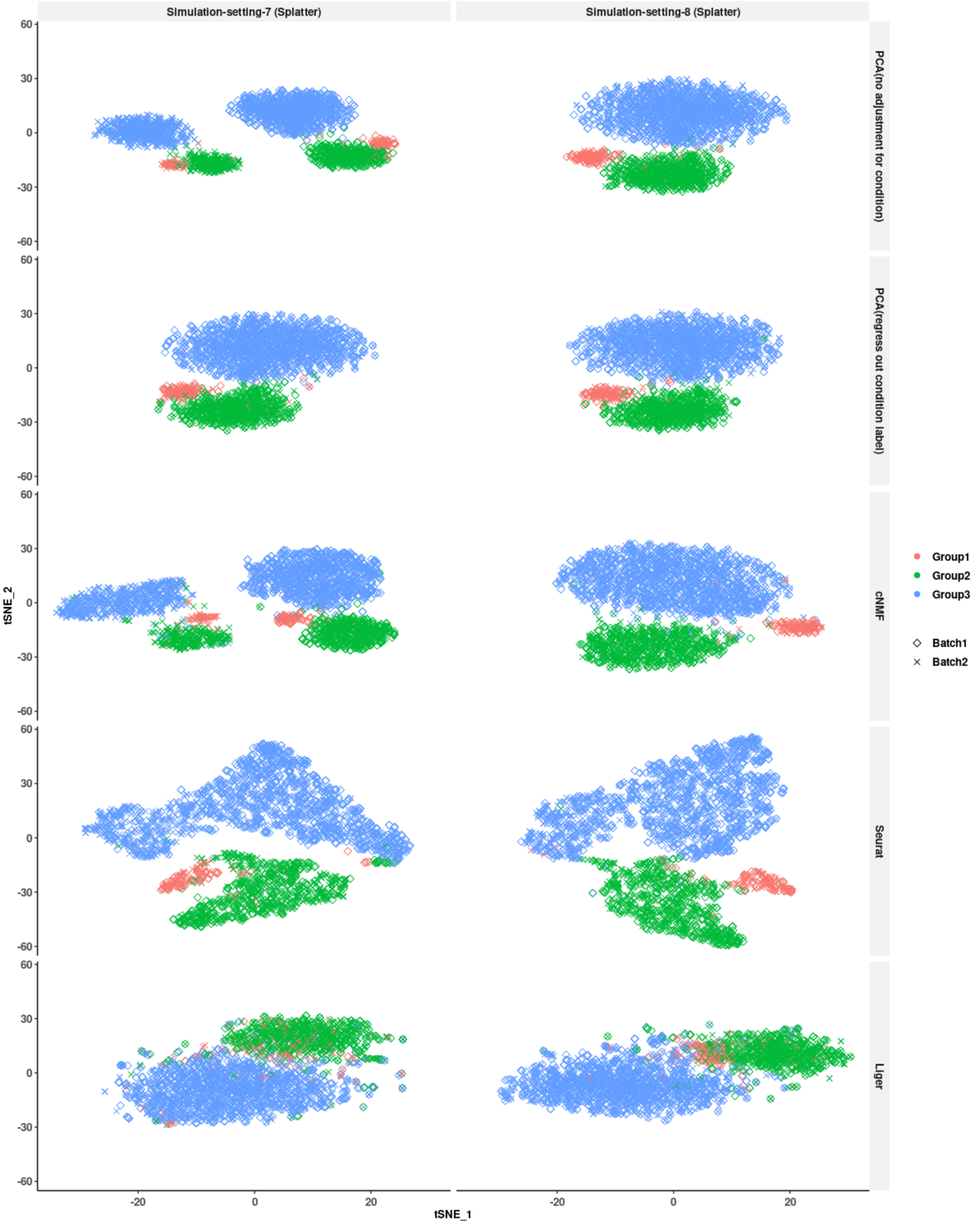

Figure S7: Visualization of a sample Splatter simulated data under the tSNE coordinates computed using multiple dimension reduction results (PCA, cNMF, Seurat, and Liger). "Simulation-setting-7" on the left represents "rare cell type and large condition effects". "Simulation- setting-8" on the right represents setting "rare cell type and small condition effects". Compared to the first row, the second row had an extra step in the preprocessing stage, which is to regress out unwanted variation, e.g. the condition label.

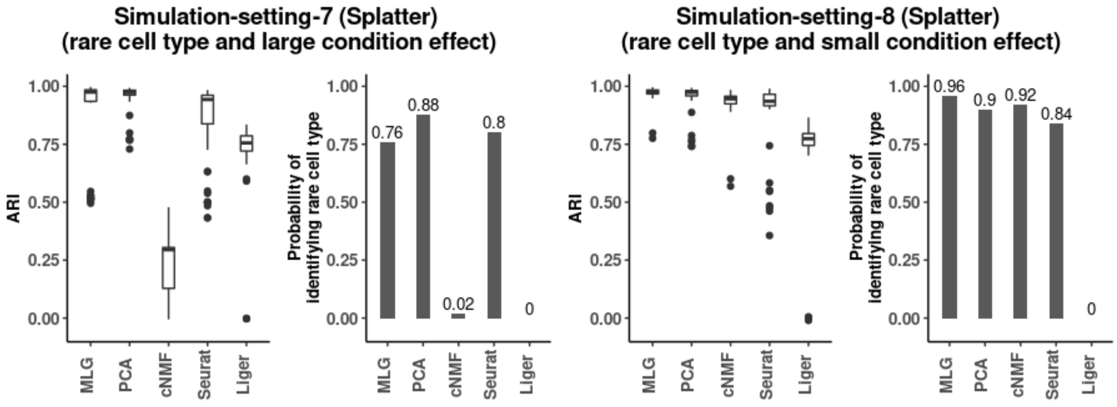

Figure S8: Clustering performance in simulation settings 7 and 8. Adjusted rand index (ARI) and rare cell type identification performances across 50 simulation replicates in simulation settings 7 and 8. PCA, cNMF, Seurat, and Liger represent Louvain clustering with the low-dimensional embeddings derived from each method. A 4-layer MLG (PCA, cNMF, Seurat, Liger) is implemented in the large condition effect setting, a 2-layer MLG (PCA, cNMF) is implemented in the small condition effect setting.

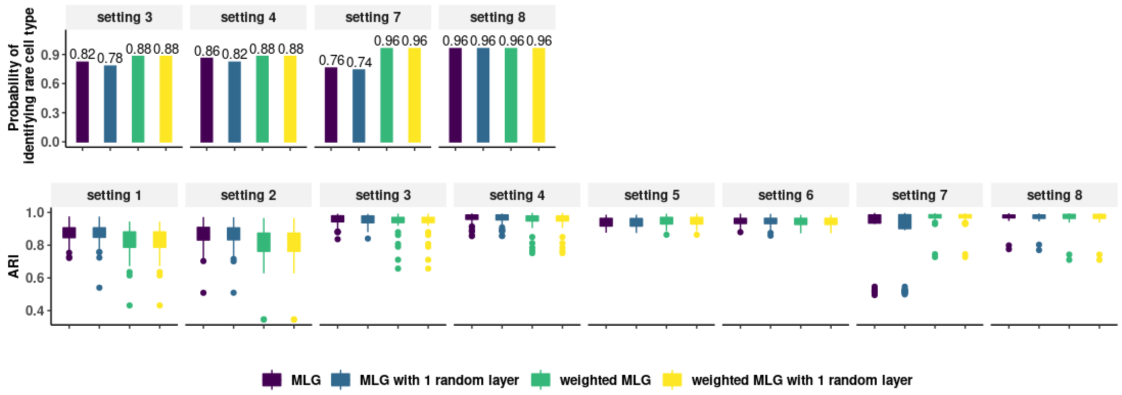

Figure S9: Performance comparison of MLG and weighted MLG with simulated datasets in setting 1-8. Rare cell type identification probability in rare cell type simulations (setting 3, 4, 7, 8) and Adjusted Rand Index in all settings are reported. A 4-layer MLG (PCA, cNMF, Seurat, Liger) is implemented in the large condition effect settings (setting 1, 3, 5, 7), a 2-layer MLG (PCA, cNMF) is implemented in the small-condition-effect settings (setting 2, 4, 6, 8). “Weighted MLG” contains 4 layers (PCA, cNMF, Seurat, Liger). “MLG with 1 random layer” and “Weighted MLG with 1 random layer” use one extra layer of randomly generated adjacency matrix to evaluate its robustness against irrelevant layers.

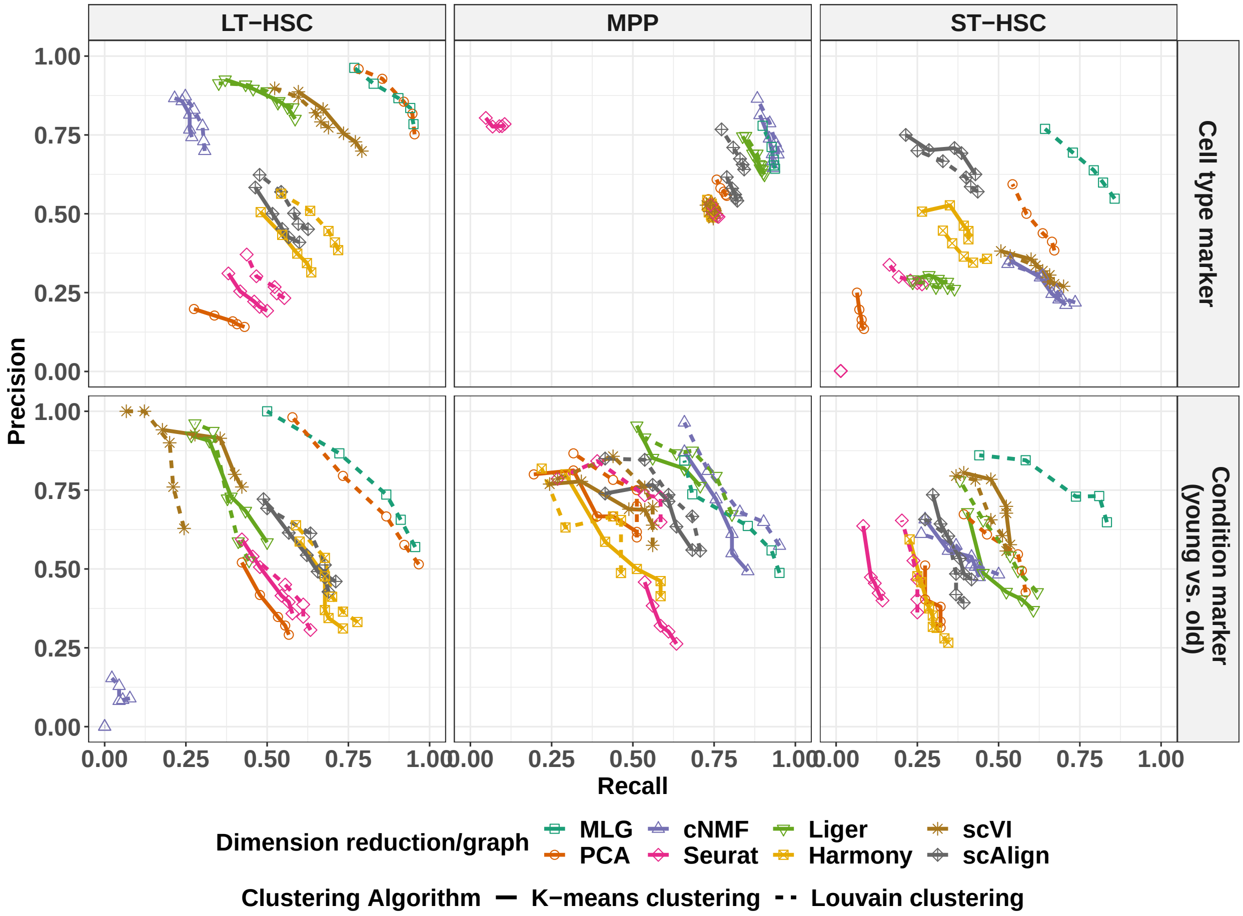

Figure S10: **Differential expression analysis for cell type/cluster marker gene and condition (young vs. old) DE gene identification in the *Kowalczyk_2* dataset.** The precision-recall (PR) values in the top panel evaluate inferred cluster (i.e., cell type) marker genes of each method against cell type marker genes defined with ground truth cell type labels as gold standard. The bottom panel evaluates the inferred condition (i.e.,age) DE genes of each cell type against the gold standard. Gold standard cell type marker and age DE genes are defined as genes with Bonferroni corrected p-values less than 0.01 in the differential expression analysis with ground truth cell labels. The PR values are reported at cutoffs of 0.2, 0.1, 0.05, 0.01, and 0.001 for Bonferroni adjusted p-values in the cluster marker gene and age DE gene identification analysis.

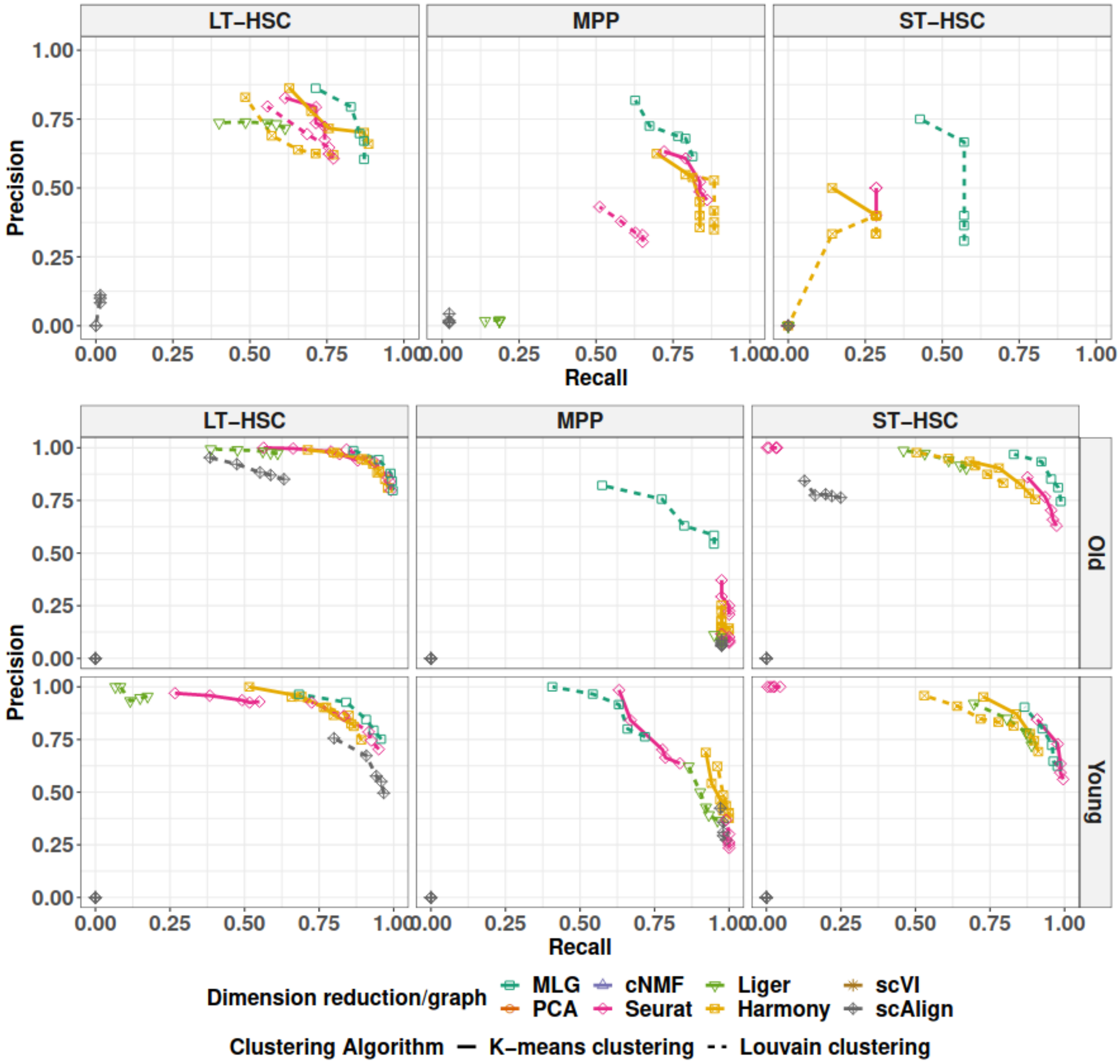

Figure S11: **Differential expression analysis for cell type/cluster marker gene and LPS+PAM stimuli DE gene identification in the *Mann* dataset.** Top panel precision-recall (PR) values evaluate inferred cluster, i.e., cell type, marker genes of each method with respect to cell type maker genes identified with ground truth cell type labels as gold standard. The bottom panel evaluates the inferred condition (i.e., LPS+PAM stimuli) DE genes of each cell type against the gold standard, separately for old and young mice. Gold standard cell type marker and LPS+PAM stimuli DE genes are defined as genes with Bonferroni corrected p-values less than 0.01 in the differential expression analysis with ground truth cell labels. The PR values are reported at cutoffs of 0.2, 0.1, 0.05, 0.01, 0.001 for Bonferroni adjusted p-values in the differential analysis for cluster marker genes and stimuli DE genes within each cluster. Several clustering of low-dimensional projections (k-means-PCA, Louvain-PCA, k-means-Liger, k-means-scVI, Louvain-scVI, k-means-cNMF, Louvain-cNMF) identified clusters with only cells from one condition, preventing a cell type-specific condition DE gene analysis.

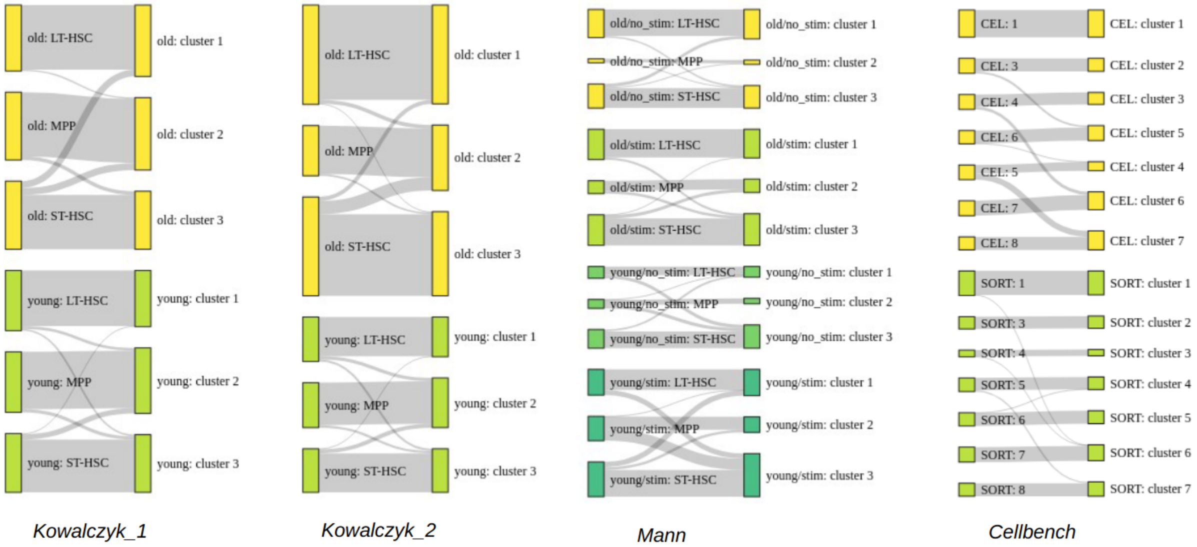

Figure S12: **River plots for the MLG results of the benchmark datasets of Figure 4.**

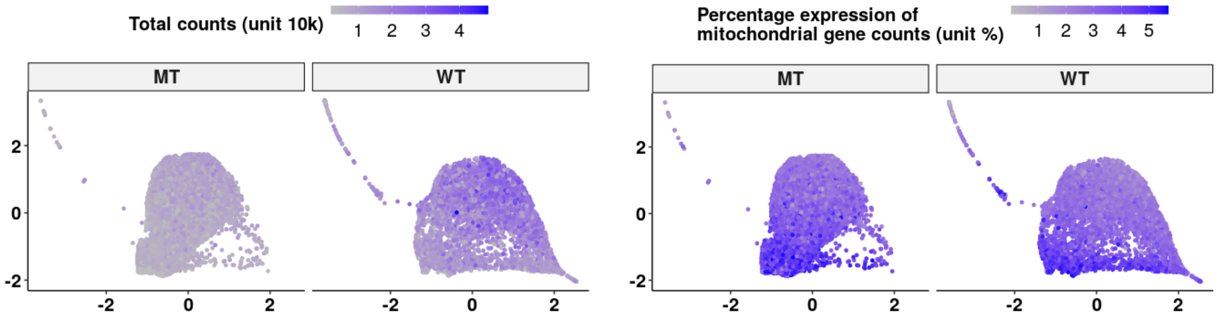

Figure S13: **SPRING visualization of the *Johnson_20* dataset, superimposed with total counts (left) and percentage expression of mitochondrial genes (right) per cell.**

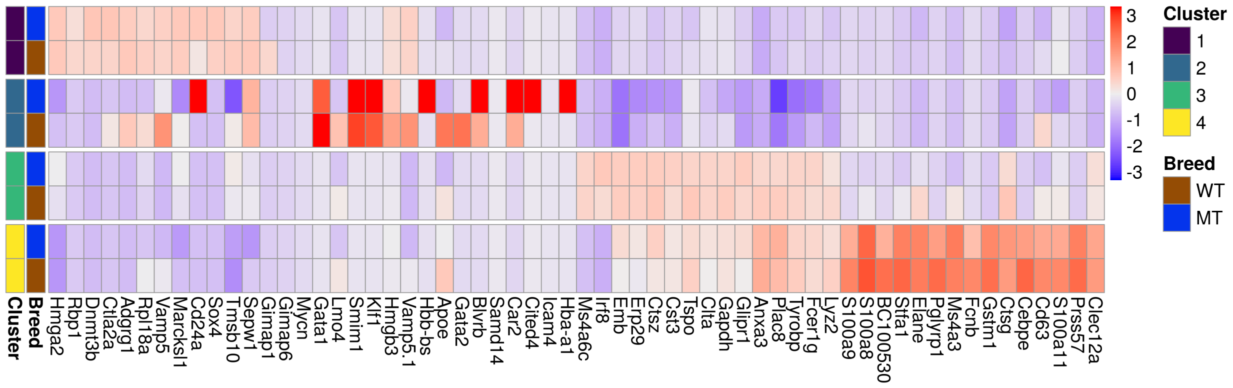

Figure S14: **Heatmap of median gene expression of MLG cluster marker genes, grouped by breed and MLG cluster, for the *Johnson_20* dataset.**

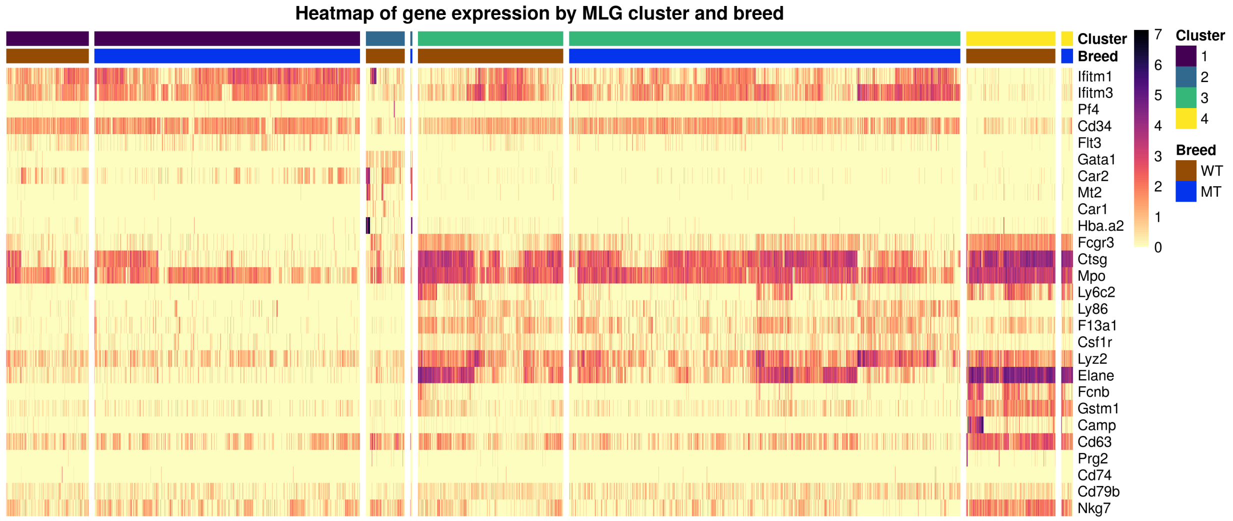

Figure S15: **Heatmap of cell-level gene expression of marker genes from [11], grouped by breed and MLG clustering of the *Johnson_20* dataset.**

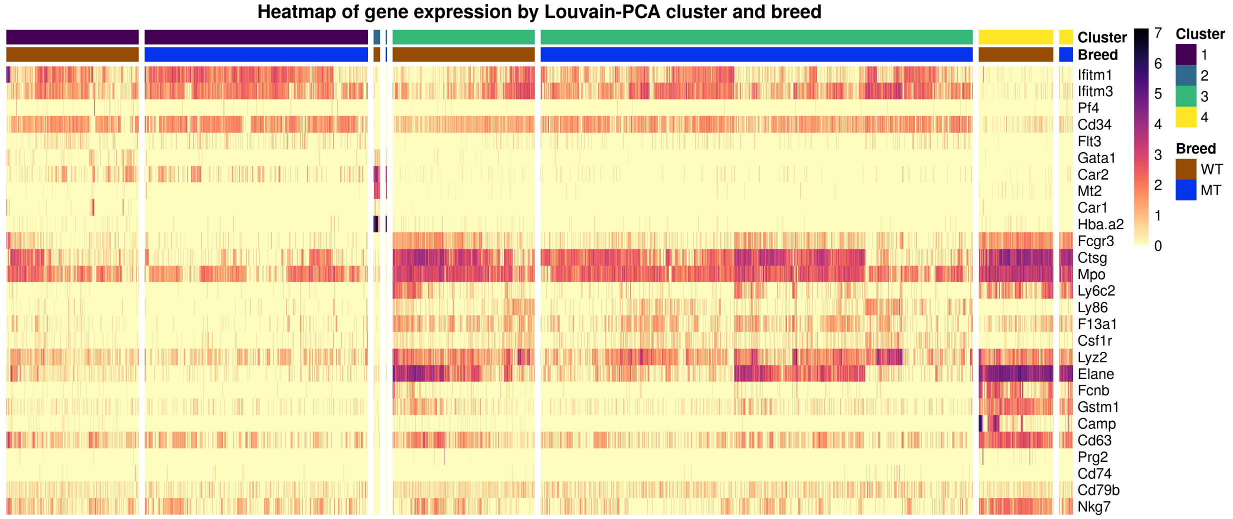

Figure S16: **Heatmap of cell-level gene expression of marker genes from [11], grouped by breed and Louvain clustering of PCA low-dimensional embedding of the *Johnson_20* dataset.**

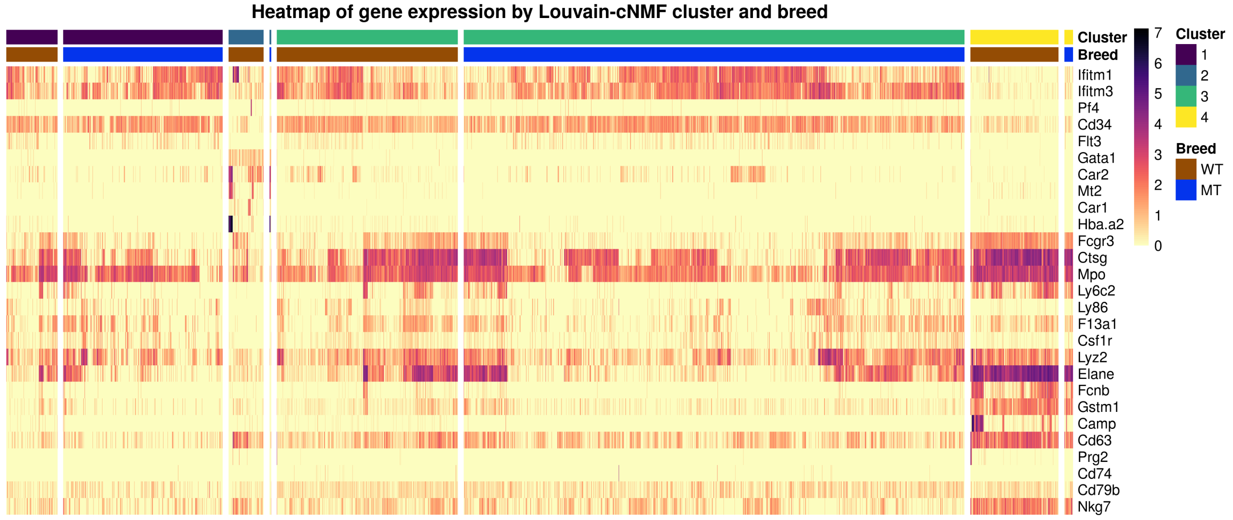

Figure S17: **Heatmap of cell-level gene expression of marker genes from [11], grouped by breed and Louvain clustering of cNMF low-dimensional embedding of the *Johnson_20* dataset.**

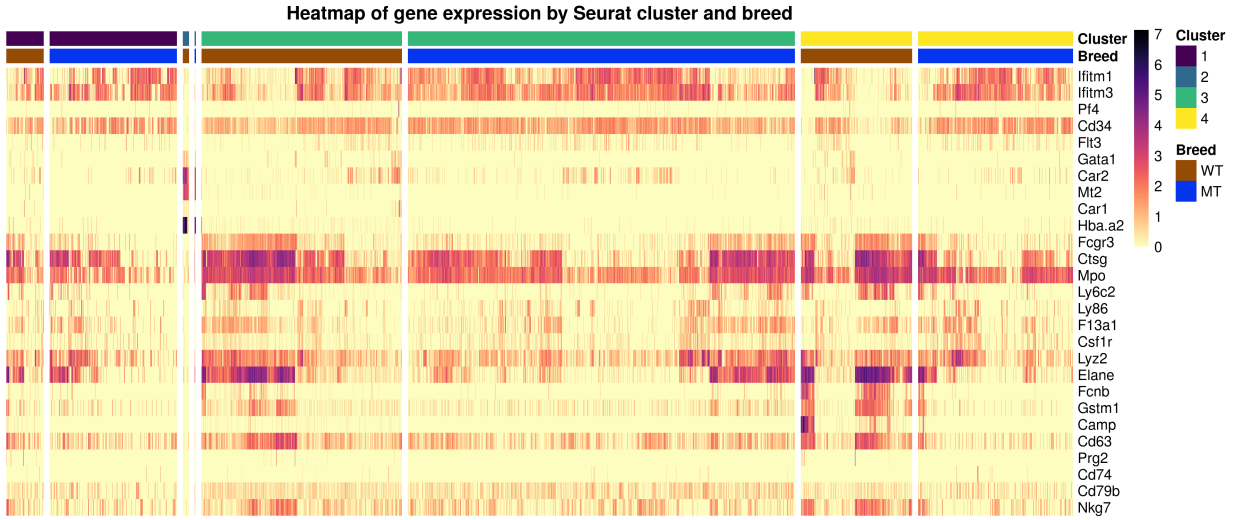

Figure S18: **Heatmap of cell-level gene expression of marker genes from [11], grouped by breed and Louvain clustering of Seurat-integration low-dimensional embedding of the *Johnson_20* dataset.**

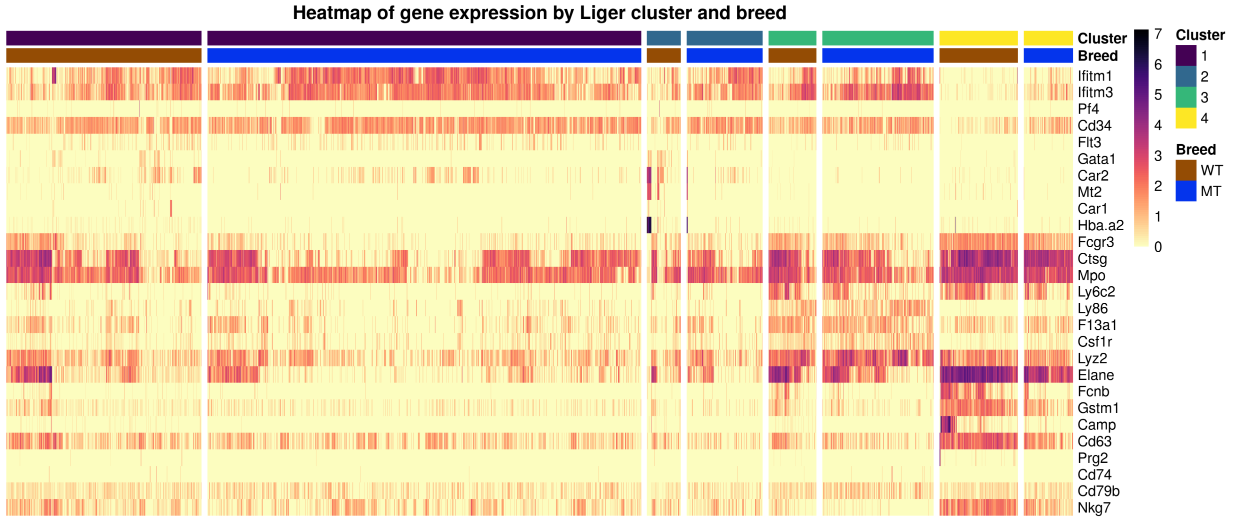

Figure S19: **Heatmap of cell-level gene expression of marker genes from [11], grouped by breed and Louvain clustering of Liger low-dimensional embedding of the *Johnson_20* dataset.**

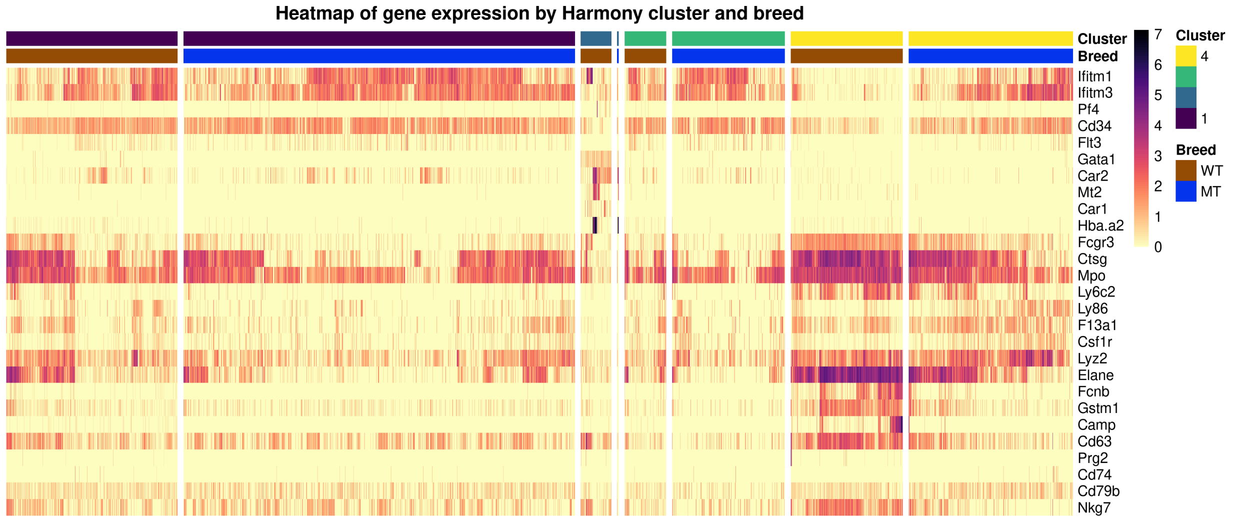

Figure S20: **Heatmap of cell-level gene expression of marker genes from [11], grouped by breed and Louvain clustering of Harmony low-dimensional embedding of the *Johnson_20* dataset.**

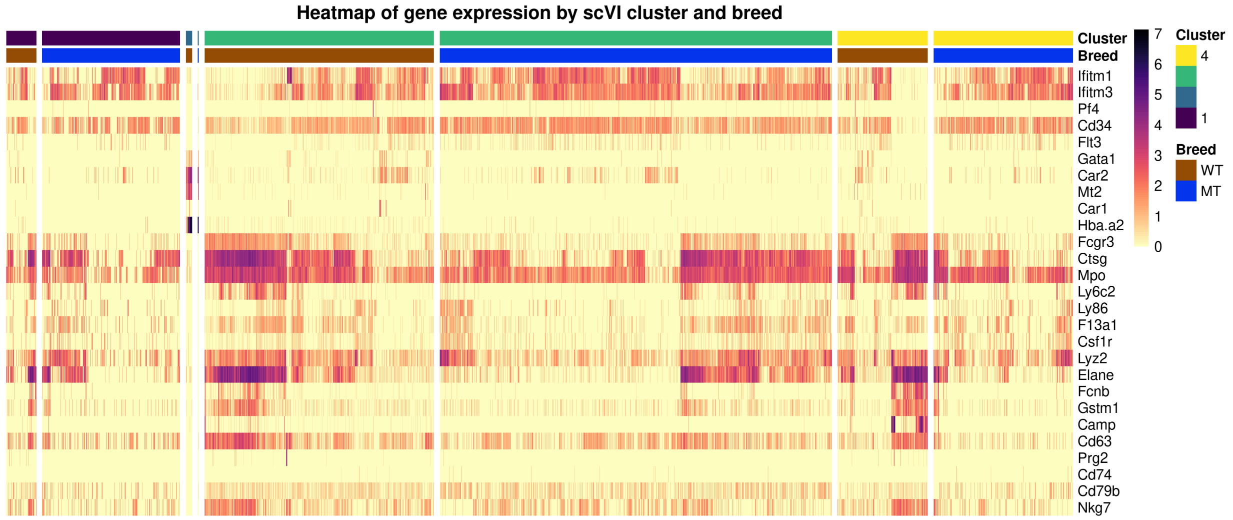

Figure S21: **Heatmap of cell-level gene expression of marker genes from [11], grouped by breed and Louvain clustering of scVI low-dimensional embedding of the *Johnson_20* dataset.**

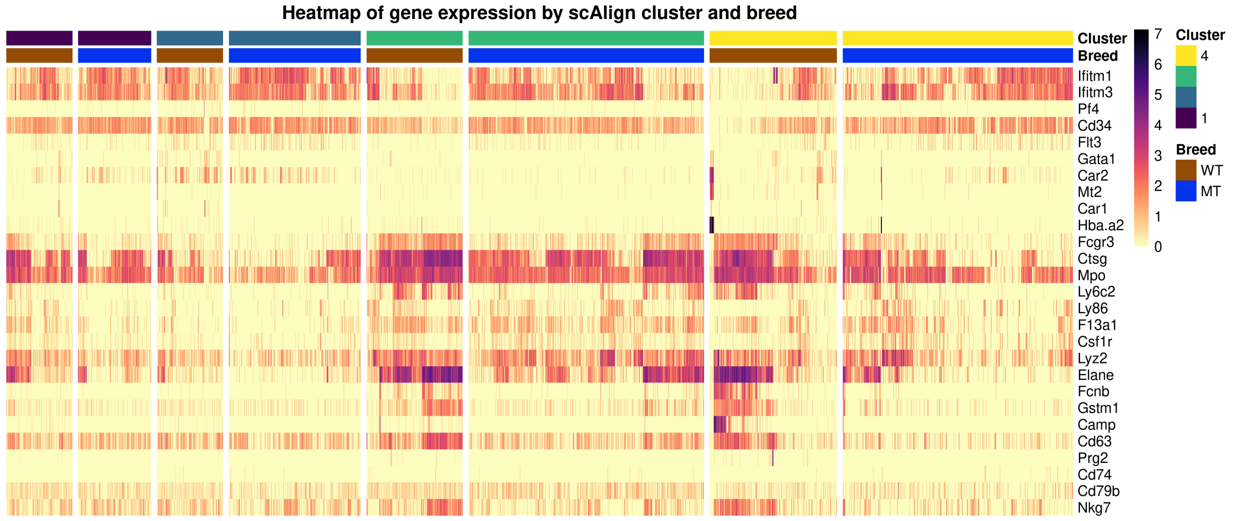

Figure S22: **Heatmap of cell-level gene expression of marker genes from [11], grouped by breed and Louvain clustering of scAlign low-dimensional embedding of the *Johnson_20* dataset.**

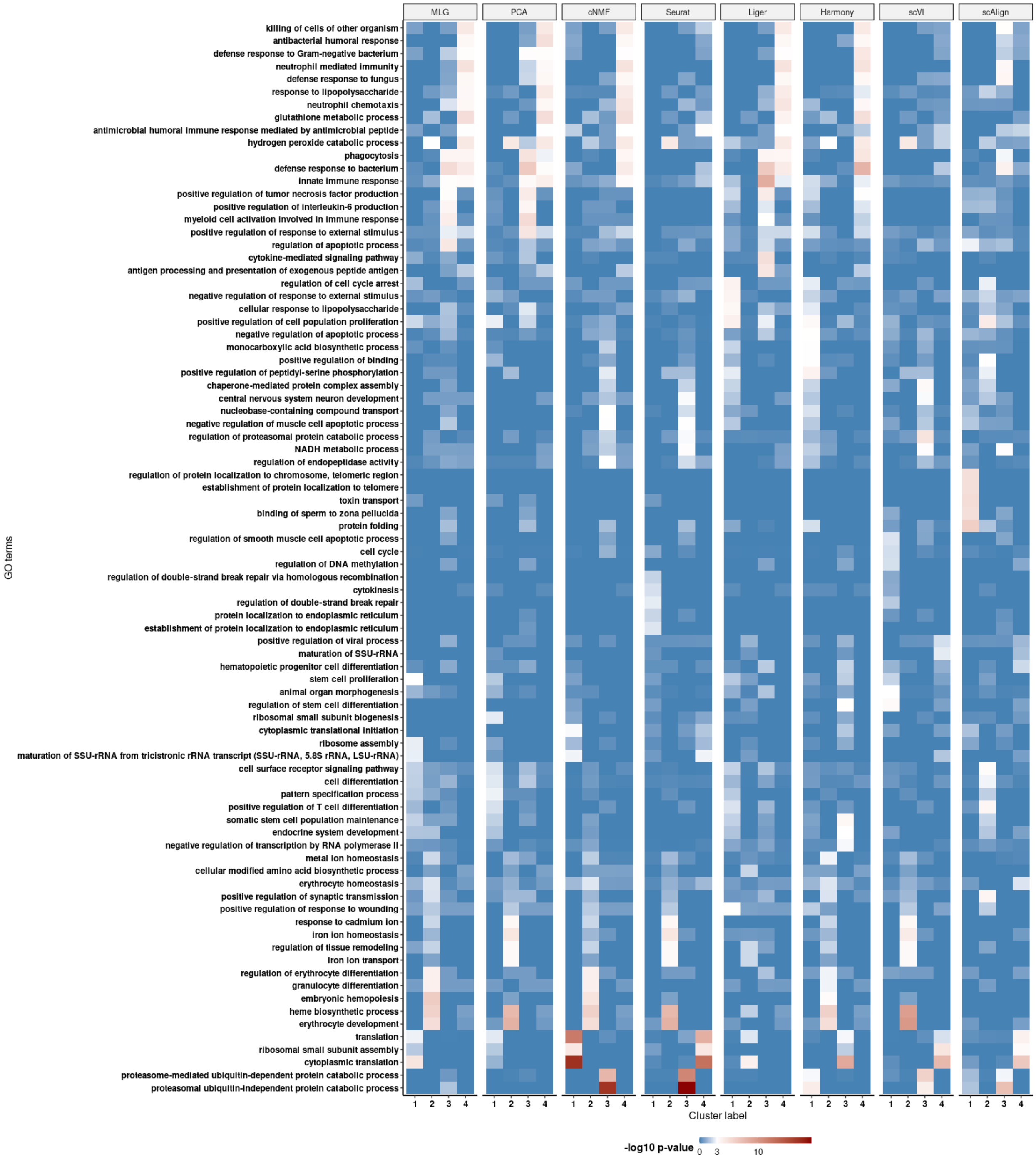

Figure S23: **Gene set enrichment analysis for the *Johnson_20* dataset.** Heatmap of the -$\log_{10}$ enrichment p-values of the GO terms across clusters identified by different methos. Top 5 GO terms from gene set enrichment analysis of each cluster of each individual method are pooled together.

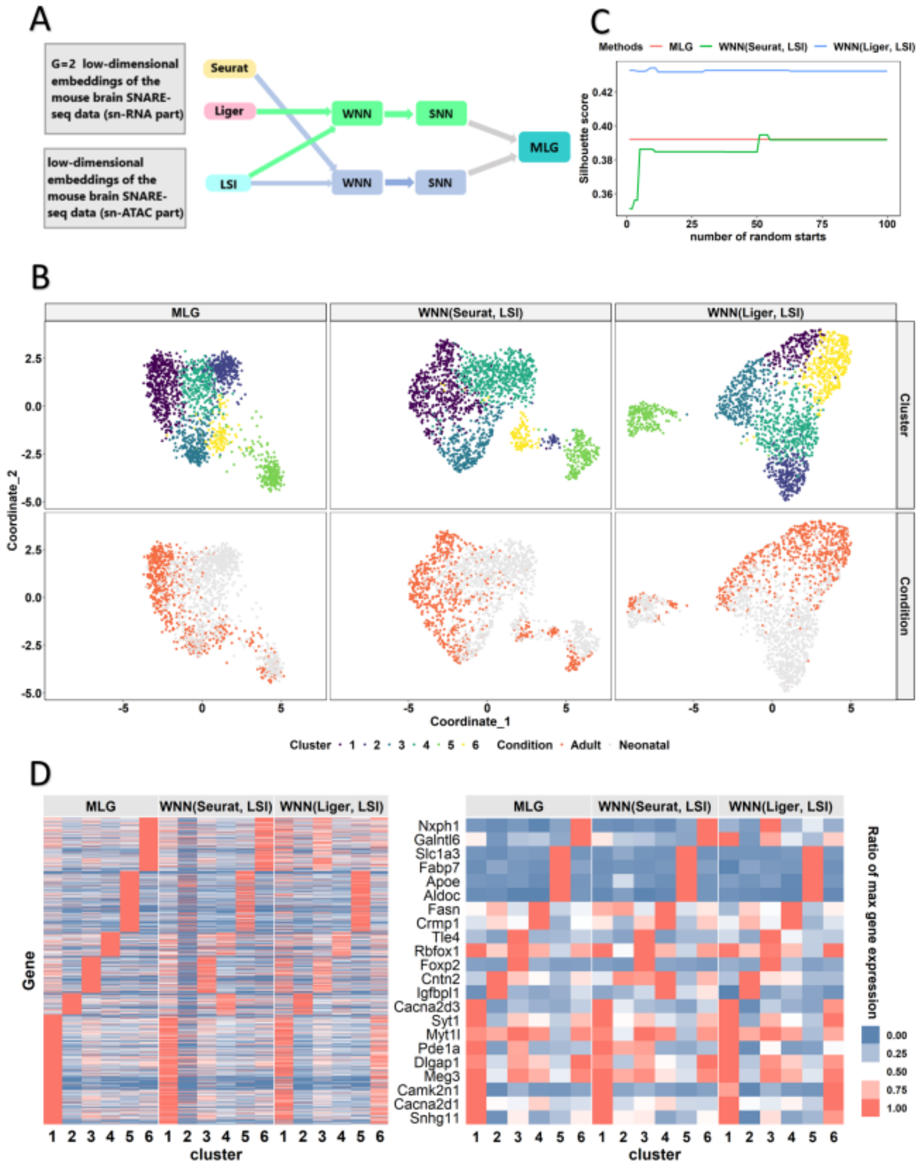

Figure S24: **Application to SNARE-Seq [6] dataset *Chen19* (replicate 1).** (A) Data processing workflow of MLG for SNARE-seq. Dimension reduction methods Liger and Seurat are applied to snRNA-seq, Latent Semantic Indexing (LSI) [4] is applied to snATAC-Seq. Weighted nearest neighbor (WNN) graphs [5] are constructed using Liger-LSI and Seurat-LSI low-dimensional embeddings. Finally, the two WNN graphs are inputted to the MLG framework. (B) Visualization of clusters and conditions on the force-directed layout of MLG and the UMAP coordinates of WNN(Seurat, LSI) and WNN(Liger, LSI). (C) Average silhouette scores for the three clustering methods, computed with the corresponding UMAP or force-directed layout coordinates. X-axis is the number of random starts inputted to the Louvain algorithm. (D) Heatmap of the scaled gene expression for all the genes (left) and selected genes (on the right).

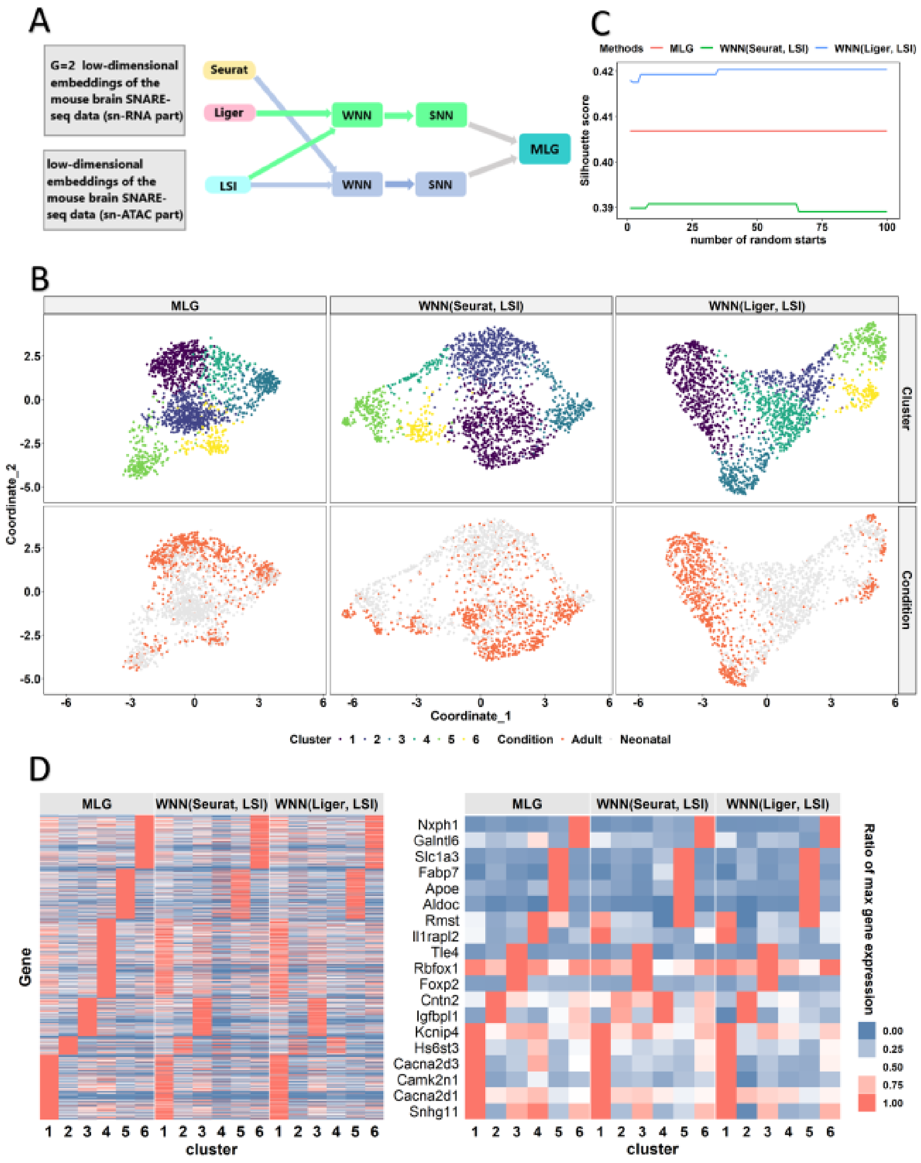

Figure S25: **Application to SNARE-Seq [6] dataset *Chen19* (replicate 2).** (A) Data processing workflow of MLG for SNARE-seq. Dimension reduction methods Liger and Seurat are applied to snRNA-seq, Latent Semantic Indexing (LSI) is applied to snATAC-Seq. Weighted nearest neighbor (WNN) graphs [5] are constructed using Liger-LSI and Seurat-LSI low-dimensional embeddings. Finally, the two WNN graphs are inputted to the MLG framework. (B) Visualization of clusters and conditions on the force-directed layout of MLG and the UMAP coordinates of WNN(Seurat, LSI) and WNN(Liger, LSI). (C) Average silhouette scores for the three clustering methods, computed with the corresponding UMAP or force-directed layout coordinates. X-axis is the number of random starts inputted to the Louvain algorithm. (D) Heatmap of the scaled gene expression for all the genes (left) and selected genes (on the right).

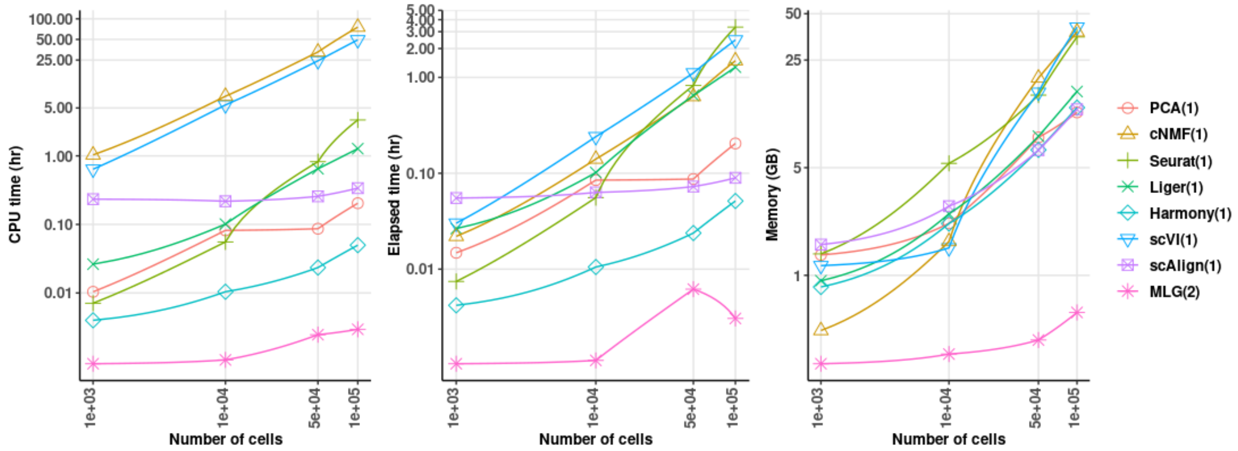

Figure S26: Time and memory usage of step (1) dimension reduction and step (2) construction of MLG graph and Louvain clustering in four datasets of 1e3, 1e4, 5e4, 1e5 cells. CPU time and elapsed time in hours, peak memory usage in gigabytes are measured by the time(1) function available in Linux systems.

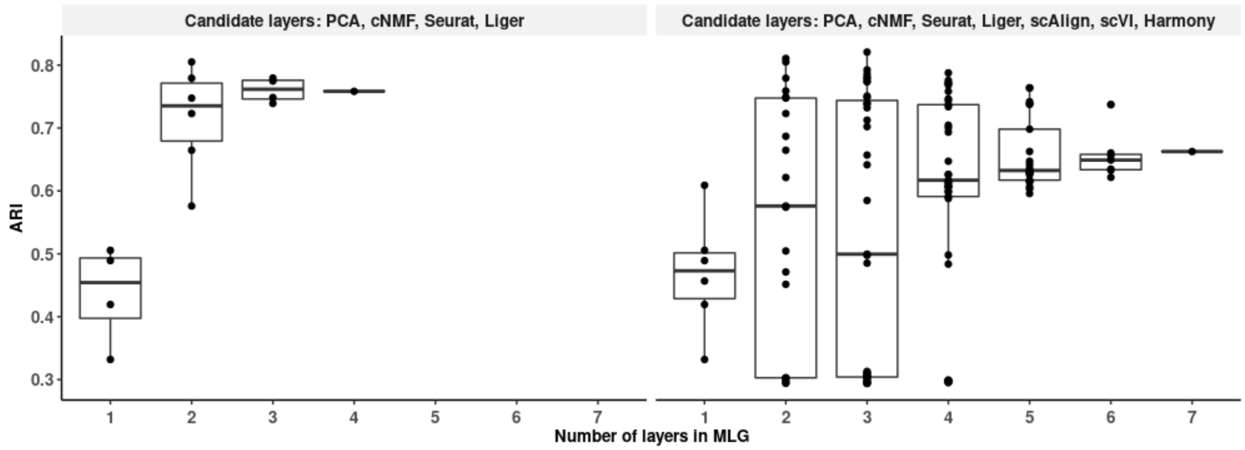

Figure S27: Evaluation of the performance of MLG with varying numbers of layers and combinations of methods using benchmark dataset *Kowalczyk_1*. Each point represents an MLG result, where x-axis denotes the number of layers and y-axis corresponds to the ARI of the resulting MLG clustering with these layers. Layers are constructed using a combination of low-dimensional embeddings from PCA, cNMF, Seurat, Liger (left), and PCA, cNMF, Seurat, Liger, scAlign, scVI, Harmony (right).

1.7 Supplementary tables

Dataset	# of cells	System	Conditions/Stimuli
Kowalczyk_1 [12]	1,058	HSPC	Young (2-3 months) and old (22 months) mice.
Kowalczyk_2 [12]	1,428	HSPC	Young (2-3 months) and old (22 months) mice.
Mann [13]	949	HSPC	Young (8-12 weeks)<br>and old (20-24 months) mice<br>with and without LPS+PAM stimulation.
Cellbench [14]	636	Synthetic cells	Two sequencing protocols:<br>CEL-seq-2 and SORT-seq.

Table S1: Benchmark datasets.

		Condition			
Kowalczyk_1	cell type	old	young		
	LT-HSC	183	167		
	MPP	188	168		
	ST-HSC	189	163		
Kowalczyk_2	cell type	old	young		
	LT-HSC	372	167		
	MPP	188	168		
	ST-HSC	370	163		
Mann	cell type	old, no stimuli	old, stimuli	young, no stimuli	young, stimuli
	LT-HSC	105	113	44	96
	MPP	15	48	33	91
	ST-HSC	90	114	70	130
Cellbench	mix proportions	CEL-seq-2	SORT-seq		
	0,0,1	44	41		
	0,1,0	45	20		
	1,0,0	45	37		
	0.16,0.16,0.68	39	39		
	0.16,0.68,0.16	45	45		
	0.68,0.16,0.16	40	40		
	0.33,0.33,0.33	82	74		

Table S2: Details on cell populations in benchmark datasets. Cell groups in the Cellbench dataset represent mixture proportion of RNA extracted from three cell lines.

Setting 1

	Cell population proportion			Activity GEP 1 usage			Activity GEP 2 usage		
cell type	1	2	3	1	2	3	1	2	3
condition 1	12.5%	25%	12.5%	0	0	5%	40%	40%	40%
condition 2	15%	15%	20%	25%	20%	20%	5%	0	0

Setting 2

	Cell population proportion			Activity GEP 1 usage			Activity GEP 2 usage		
cell type	1	2	3	1	2	3	1	2	3
condition 1	12.5%	25%	12.5%	5%	10%	5%	30%	25%	20%
condition 2	15%	15%	20%	30%	25%	25%	5%	0	10%

Setting 3

	Cell population proportion			Activity GEP 1 usage			Activity GEP 2 usage		
cell type	1	2	3	1	2	3	1	2	3
condition 1	2%	12%	26%	0	0	5%	40%	40%	40%
condition 2	3%	18%	39%	25%	20%	20%	5%	0	0

Setting 4

	Cell population proportion			Activity GEP 1 usage			Activity GEP 2 usage		
cell type	1	2	3	1	2	3	1	2	3
condition 1	2%	12%	26%	5%	10%	5%	30%	25%	20%
condition 2	3%	18%	39%	30%	25%	25%	5%	0	10%

Table S3: Parameters in simulation settings 1-4. Parameters generating identity GEPs are ($\pi = 0.025$, $\pi^d = 0.2$, $\mu = 0.65$, $\sigma = 0.25$) in setting 1 & 2 and ($\pi = 0.025$, $\pi^d = 0.2$, $\mu = 0.85$, $\sigma = 0.25$) in setting 3 & 4. Parameters generating activity GEPs are ($\pi = 0.05$, $\pi^d = 0.2$, $\mu = 0.7$, $\sigma = 0.25$) in all 4 settings.

Setting 5

`batch.facLoc`	`batch.facScale`	`group.prob`	`de.prob`	`de.downProb`	`de.facLoc`	`de.facScale`
0.2	0.05	(0.2, 0.3, 0.5)	0.025	0.2	0.5	0.25

Setting 6

0.025	0.025	(0.2, 0.3, 0.5)	0.025	0.2	0.5	0.25

Setting 7

0.2	0.05	(0.05, 0.3, 0.65)	0.025	0.2	0.6	0.25

Setting 8

0.025	0.025	(0.05, 0.3, 0.65)	0.025	0.2	0.6	0.25

Table S4: Splatter Parameters in Simulation settings 5-8. Parameters that are common for all Splatter simulation settings are as follows `mean.shape` 1.46, `mean.rate` 1.48, `lib.loc` 8.95, `lib.scale` 0.45, `out.prob` 0.091, `out.facLoc` 2.82, `out.facScale` 0.84, `bcv.common` 0.11, `bcv.df` 36.57, `batchCells` (1500, 1000), `nGenes` 3000.

	All genes		
Method	MLG	WNN(Seurat, LSI)	WNN(Liger, LSI)
Chisq Statistic	156,864.8	131,409.0	114,835.7
	Marker genes		
Method	MLG	WNN(Seurat, LSI)	WNN(Liger, LSI)
Chisq Statistic	3,118.87	2,583.21	2,072.69

Table S5: Chi-squared statistic from Fisher's combined probability test for the genes in Supplementary Figure S24.

	All genes		
Method	MLG	WNN(Seurat, LSI)	WNN(Liger, LSI)
Chisq Statistic	102,058.2	111,937.6	144,229.7
	Marker genes		
Method	MLG	WNN(Seurat, LSI)	WNN(Liger, LSI)
Chisq Statistic	1,400.01	1,613.70	2,433.14

Table S6: Chi-squared statistic from Fisher's combined probability test for the genes in Supplementary Figure S25.

Method	MLG	PCA	cNMF	Seurat	Liger	Harmony	scVI	scAlign
Chisq Statistic	33,746.46	31,660.67	27,404.00	4,632.68	33,198.90	24,061.36	3,878.32	13,140.07

Table S7: Chi-squared statistic from Fisher's combined probability test for the genes in Supplementary Figure S15-S22.

References

[1] Paul W Holland, Kathryn Blackmond Laskey, and Samuel Leinhardt. Stochastic blockmodels: First steps. *Social networks*, 5(2):109–137, 1983.

[2] Anderson Y Zhang, Harrison H Zhou, et al. Minimax rates of community detection in stochastic block models. *The Annals of Statistics*, 44(5):2252–2280, 2016.

[3] Rick Durrett. *Probability: theory and examples*, volume 49. Cambridge university press, 2019.

[4] Darren A Cusanovich, Riza Daza, Andrew Adey, Hannah A Pliner, Lena Christiansen, Kevin L Gunderson, Frank J Steemers, Cole Trapnell, and Jay Shendure. Multiplex single-cell profiling of chromatin accessibility by combinatorial cellular indexing. *Science*, 348(6237):910–914, 2015.

[5] Yuhan Hao, Stephanie Hao, Erica Andersen-Nissen, William M Mauck III, Shiwei Zheng, Andrew Butler, Maddie J Lee, Aaron J Wilk, Charlotte Darby, Michael Zager, et al. Integrated analysis of multimodal single-cell data. *Cell*, 2021.

[6] Song Chen, Blue B Lake, and Kun Zhang. High-throughput sequencing of the transcriptome and chromatin accessibility in the same cell. *Nature biotechnology*, 37(12):1452–1457, 2019.

[7] Zhanying Feng, Xianwen Ren, Yuan Fang, Yining Yin, Chutian Huang, Yimin Zhao, and Yong Wang. sctim: seeking cell-type-indicative marker from single cell rna-seq data by consensus optimization. *Bioinformatics*, 36(8):2474–2485, 2020.

[8] John D Cahoy, Ben Emery, Amit Kaushal, Lynette C Foo, Jennifer L Zamanian, Karen S Christopherson, Yi Xing, Jane L Lubischer, Paul A Krieg, Sergey A Krupenko, et al. A transcriptome database for astrocytes, neurons, and oligodendrocytes: a new resource for understanding brain development and function. *Journal of Neuroscience*, 28(1):264–278, 2008.

[9] Hannah Hochgerner, Amit Zeisel, Peter Lönnerberg, and Sten Linnarsson. Conserved properties of dentate gyrus neurogenesis across postnatal development revealed by single-cell rna sequencing. *Nature neuroscience*, 21(2):290–299, 2018.

[10] Ying-Jiun J Chen, Brad A Friedman, Connie Ha, Steffen Durinck, Jinfeng Liu, John L Rubenstein, Somasekar Seshagiri, and Zora Modrusan. Single-cell rna sequencing identifies distinct mouse medial ganglionic eminence cell types. *Scientific reports*, 7(1):1–11, 2017.

[11] Amir Giladi, Franziska Paul, Yoni Herzog, Yaniv Lubling, Assaf Weiner, Ido Yofe, Diego Jaitin, Nina Cabezas-Wallscheid, Regine Dress, Florent Ginhoux, et al. Single-cell characterization of haematopoietic progenitors and their trajectories in homeostasis and perturbed haematopoiesis. *Nature cell biology*, 20(7):836–846, 2018.

[12] Monika S Kowalczyk, Itay Tirosh, Dirk Heckl, Tata Nageswara Rao, Atray Dixit, Brian J Haas, Rebekka K Schneider, Amy J Wagers, Benjamin L Ebert, and Aviv Regev. Single-cell rna-seq reveals changes in cell cycle and differentiation programs upon aging of hematopoietic stem cells. *Genome research*, 25(12):1860–1872, 2015.

[13] Mati Mann, Arnav Mehta, Carl G de Boer, Monika S Kowalczyk, Kevin Lee, Pearce Haldeman, Noga Rogel, Abigail R Knecht, Daneyal Farouq, Aviv Regev, et al. Heterogeneous responses of hematopoietic stem cells to inflammatory stimuli are altered with age. *Cell reports*, 25(11): 2992–3005, 2018.

[14] Luyi Tian, Xueyi Dong, Saskia Freytag, Kim-Anh Le Cao, Shian Su, Abolfazl JalalAbadi, Daniela Amann-Zalcenstein, Tom S Weber, Azadeh Seidi, Jafar S Jabbari, et al. Benchmarking single cell rna-sequencing analysis pipelines using mixture control experiments. *Nature methods*, 16(6):479–487, 2019.
